# Exploring the Potential of Homologous Recombination Protein PALB2 in Synthetic Lethal Combinations

**DOI:** 10.1021/acschembio.5c00111

**Published:** 2025-04-29

**Authors:** Xinyan Lu, Basilius Sauter, Aramis Keller, Saule Zhanybekova, Dennis Gillingham

**Affiliations:** Department of Chemistry, University of Basel, 4056 Basel, Switzerland

## Abstract

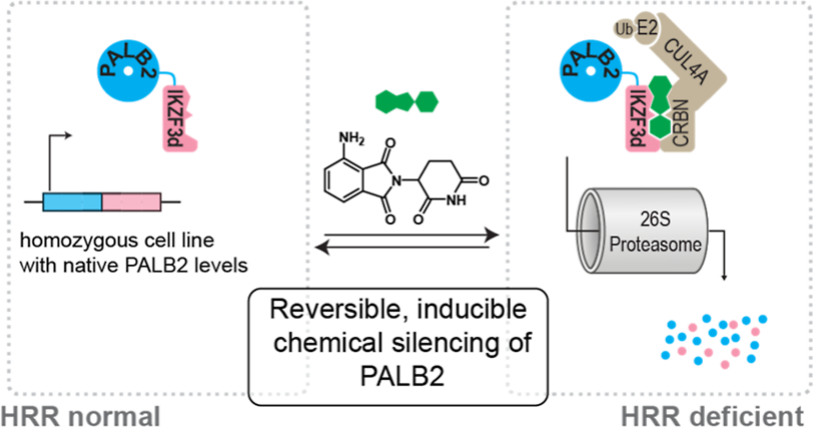

Cells with defective homologous recombination (HR) are highly sensitive to poly(ADP-ribose) polymerase (PARP) inhibition. Current therapeutic approaches leverage this vulnerability by using PARP inhibitors in cells with genetically compromised HR. However, if HR factors in cancer cells could be inhibited or degraded pharmacologically, it might reveal other opportunities for synergistic combinations. In this study, we developed a model system that recapitulates PARP/HR synthetic lethality by integrating a small-molecule responsive zinc-finger degron into the HR factor Partner and Localizer of BRCA2 (PALB2). We further tested a series of peptide ligands for PALB2 based on its natural binding partners, which led to the discovery of a high affinity peptide that will support future work on PALB2 and HR. Together, our findings validate PALB2 as a promising drug target and provide the tools and starting points for developing molecules with therapeutic applications.

## Introduction

**Figure 1:**
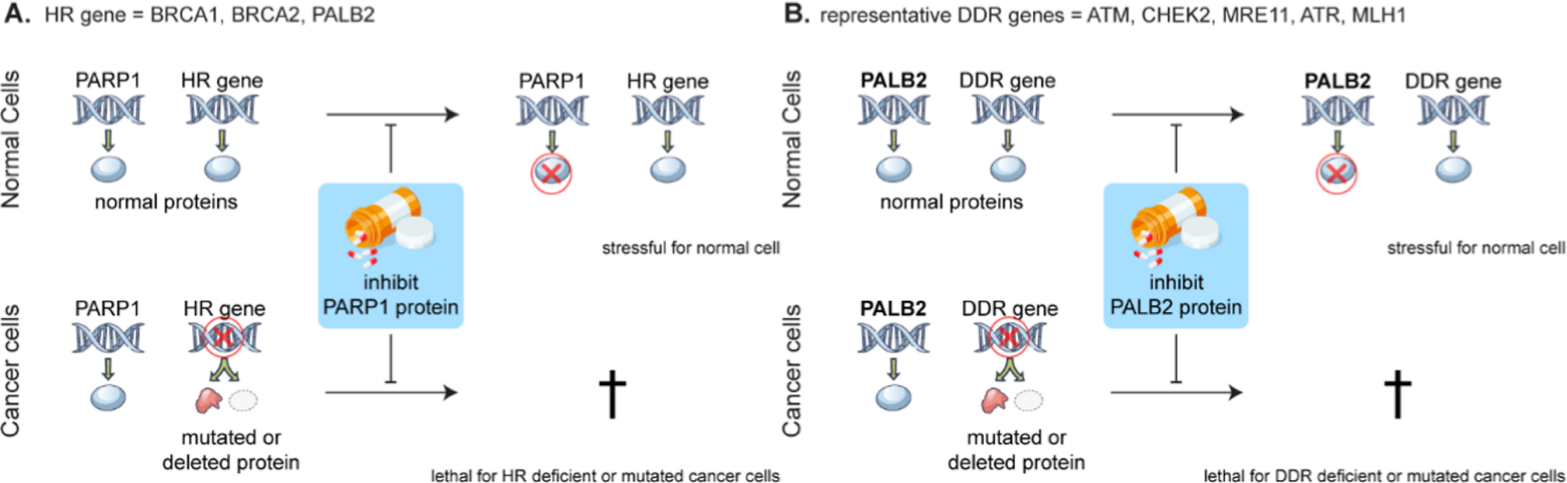
(A) Concept of synthetic lethality illustrated using the PARP1/homologous recombination deficient (HRD) synthetic lethal pair. (B) How PALB2 inhibition or depletion could create opportunities to use synthetic lethality in cancer cells that do not have HRD, but harbor mutations in other DNA-damage response (DDR) proteins.

Mutations in certain genes or their protein products can leave cells reliant on alternative or compensatory pathways for survival. Pharmacologically inhibiting these alternative pathways can be lethal to such cells, a concept known as synthetic lethality (Figure 1A).^1,2^ While synthetic lethal gene/gene or gene/drug interactions have long been recognized,^2,3^ the success of PARP inhibitors in homologous recombination-deficient (HRD) cells^4^ has renewed interest in exploring novel synthetic lethal pairs.^5,6^ Although it was PARP inhibition in patients with early onset breast cancer genes 1 and 2 (BRCA1 and BRCA2) that laid the foundation in this field,^4^ recent efforts have expanded the reach of PARP inhibitors to other genes in involved in genome maintenance.^7,8^ Hence most work in the field has focused on exploring new opportunities with PARP inhibitors^9^ in HRD or DNA damage repair (DDR)-deficient^10^ contexts.^11^ Here, however, we examine a different approach that attempts to exploit sensitivities that emerge upon depletion of the HR factor partner and localizer of BRCA2 (PALB2). PALB2 is an essential component of HR and its mutation or loss shows the same spectrum of sensitivities as BRCA1/BRCA2 loss.^7,12−15^ In contrast to BRCA1/BRCA2 however (at least at their current level of characterization), PALB2 has a WD40 β-propeller domain that is well-folded and critical for its interaction with BRCA2,^16^ which suggests PALB2 might be amenable to drug targeting.^17^ Attempts to drug HR have thus far focused exclusively on targeting Rad51, but well validated chemical matter against Rad51 has proven challenging.^18,19^ We were intrigued by the possibility of PALB2 binders both to create tool compounds that could facilitate the study of HR, but also potentially as starting points for drug development (see Figure 1B for concept). Although HR is an important DNA repair pathway, the relatively frequent occurrence of heterozygous mutations,^20^ and the existence of homozygous carriers in the form of Fanconi anemia subtypes D2 (BRCA2) and N (PALB2),^21,22^ suggest that chemically inducing HRD would be tolerated in humans. Here we develop two important tools that will facilitate studying PALB2 biology and in finding chemical matter for targeting it. The first tool is a cellular model of PALB2 loss (and an isogenic control), which enables careful study of synthetic lethal combinations of PALB2; and the second is a biochemical assay that enables the study of molecular interactions with PALB2 while potentially also giving a starting point for inhibitors.

## Results

### Systematic Analysis of the PALB2/BRCA2 Protein–Protein Interaction Leads to High-Affinity Peptide

**Figure 2:**
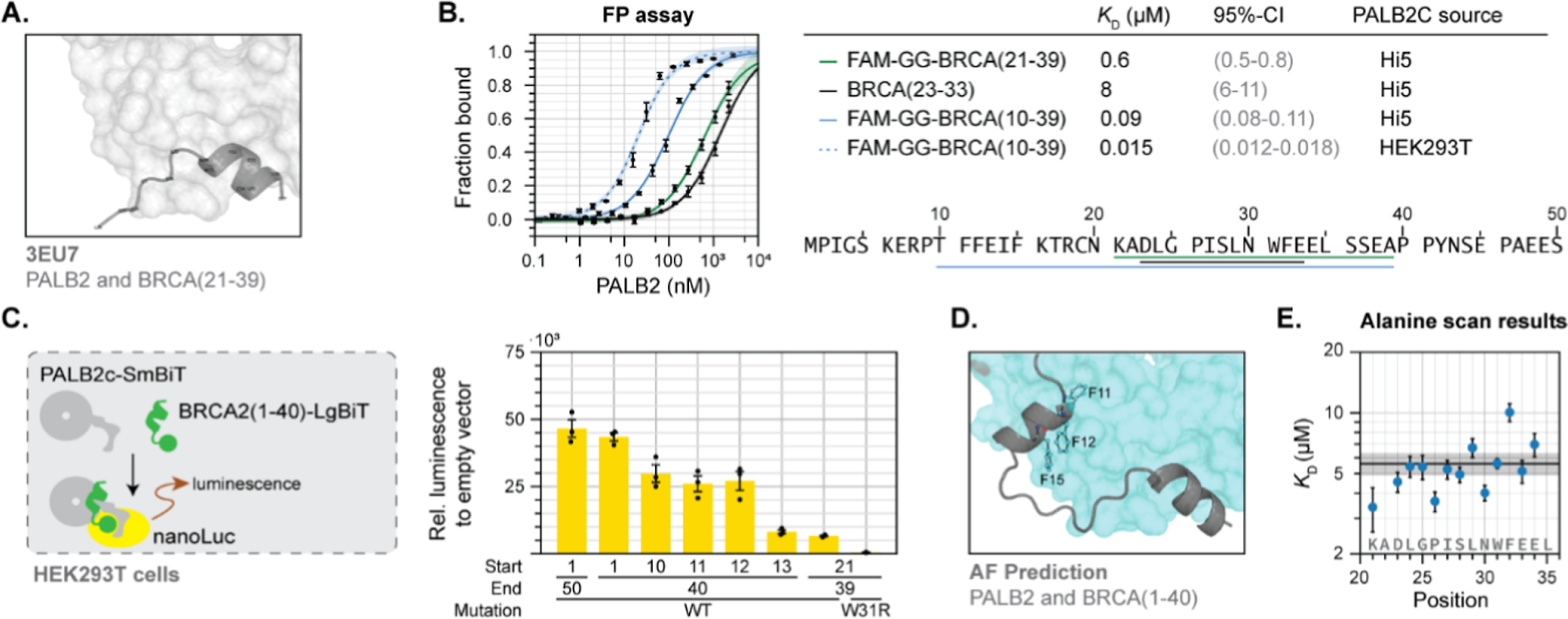
(A) Structure of BRCA2 and PALB2 (PDB: 3EU7) interaction;^16^ (B). Fluorescence polarization measurements of the BRCA2 peptide fragment’s binding with PALB2. Dissociation constant and confidence intervals, as well as the sequence of BRCA2(1–50) and the span of the three peptides are given left of it. (C) Cellular nanobit assay confirms that the PALB2/BRCA2 peptide interaction is maintained in cells and the relative interaction strength is consistent with the results of the biochemical binding assay. (D) Alphafold model of the BRCA(1–40) predicts a second important binding interaction. (E) Alanine scan from positions 21–34 of the BRCA2 peptide.

Previous structural work on the PALB2 C-terminal WD40 domain identified a small fragment of BRCA2 (residues 21–39) with an approximately 600 nM binding affinity (600 nM) to PALB2 (see Figure 2A for structure).^16^ To identify more potent binders, we first expressed the PALB2 C-terminal domain in insect cells as described previously.^16^ Binding of the BRCA2(21–39) peptide using fluorescence polarization (FP) with fluorescein (FAM) indeed gave a value in close alignment with the previous report (Figure 2B).^16^ Building from this observation we further characterized the binding of amino acids 1–40 from the BRCA2 N-terminus by first truncating bits from the N- and C-termini to determine the optimal binding peptide length. The main finding from these studies was that the first 11 N-terminal residues had only a 2-fold impact on binding. However, the residues after and leading up to twenty-one (i.e., 12–21) proved quite important, adding 6–10-fold in binding affinity (compare FAM-GG-BRCA(21–39) with FAM-GG-BRCA(10–39) in Figure 2B). The absolute minimal peptide that still gave reasonable binding was BRCA(23–33), which bound with 8 μM *K*_d_ (all measurements available in Section S8).

With biochemical binding established we wanted to verify that the peptide/PALB2 interactions were relevant in cells. Hence we developed a nanobit protein–protein interaction assay^23^ by appending a small BiT (SmBiT) to the PALB2 N-terminus and a large BiT (LgBiT) to the BRCA2 peptide C-terminus (Figure 2C). Reconstitution of the nanoLuc luciferase and release of light only occurs if these proteins interact. The results in cells are in good alignment with the biochemical experiments, since here as well truncating beyond residue 12 from the N-terminus severely compromises the binding signal. Although the BRCA(21–39) peptide still shows a signal in the cellular assay (second to last column in Figure 2C), its binding seems far weaker than the longer and full-length peptides. It should be noted, however, that biochemical binding assays suggest subtle differences in peptide binding between the PALB2 obtained from insect cell expression and that from HEK cell expression (compare blue solid and dashed traces in Figure 2B), so the absolute quantitation should be interpreted with caution. Finally a W31R mutation of the BRCA(21–39) peptide leads to complete loss of the interaction signal (final data column in Figure 2C). Some relevant missense variants of PALB2 have been identified,^16,24^ and of these we tested specifically PALB2(A1025R), which impairs the signal as well (Figure S2C). Since both the biochemical and cellular assays showed improved binding with peptides containing more of the N-terminal residues, we modeled the entire 1–40 residue BRCA2 peptide interaction with PALB2 using Alphafold.^25,26^ Interestingly, alphafold suggests that there is a second hydrophobic binding patch capable of interacting with the phenylalanine rich region of BRCA2 peptides between residues 11–15 (Figure 2D). Finally, we carried out an alanine scan to determine which residues have the strongest contribution to binding (Figure 2E). Although no single residue stands out, the hydrophobic residue F32A pointing toward the protein surface shows the strongest effect.

With tight-binding inhibitors identified we then pursued strategies to deliver these to cells. Despite efforts to deliver them by electroporation or by appending cell-penetrating motifs, we could not deliver enough peptide to compromise HR (as measured by Rad51 foci assay). Hence although the biochemical and cellular protein–protein interaction (PPI) assays we introduce in Figure 2 are useful for testing PALB2 binders or exploring variants of unknown significance in PALB2 pathologies, these molecules cannot be used to study the impact of inhibiting wild-type PALB2 in cells until we solve the delivery problem. In the absence of a cell-permeable ligand, we therefore turned to the possibility of editing the native PALB2 locus to integrate a peptide sequence that stimulates protein degradation in the presence of a specific small molecule.

### Cellular Model for Inducible HR Deficiency

**Figure 3:**
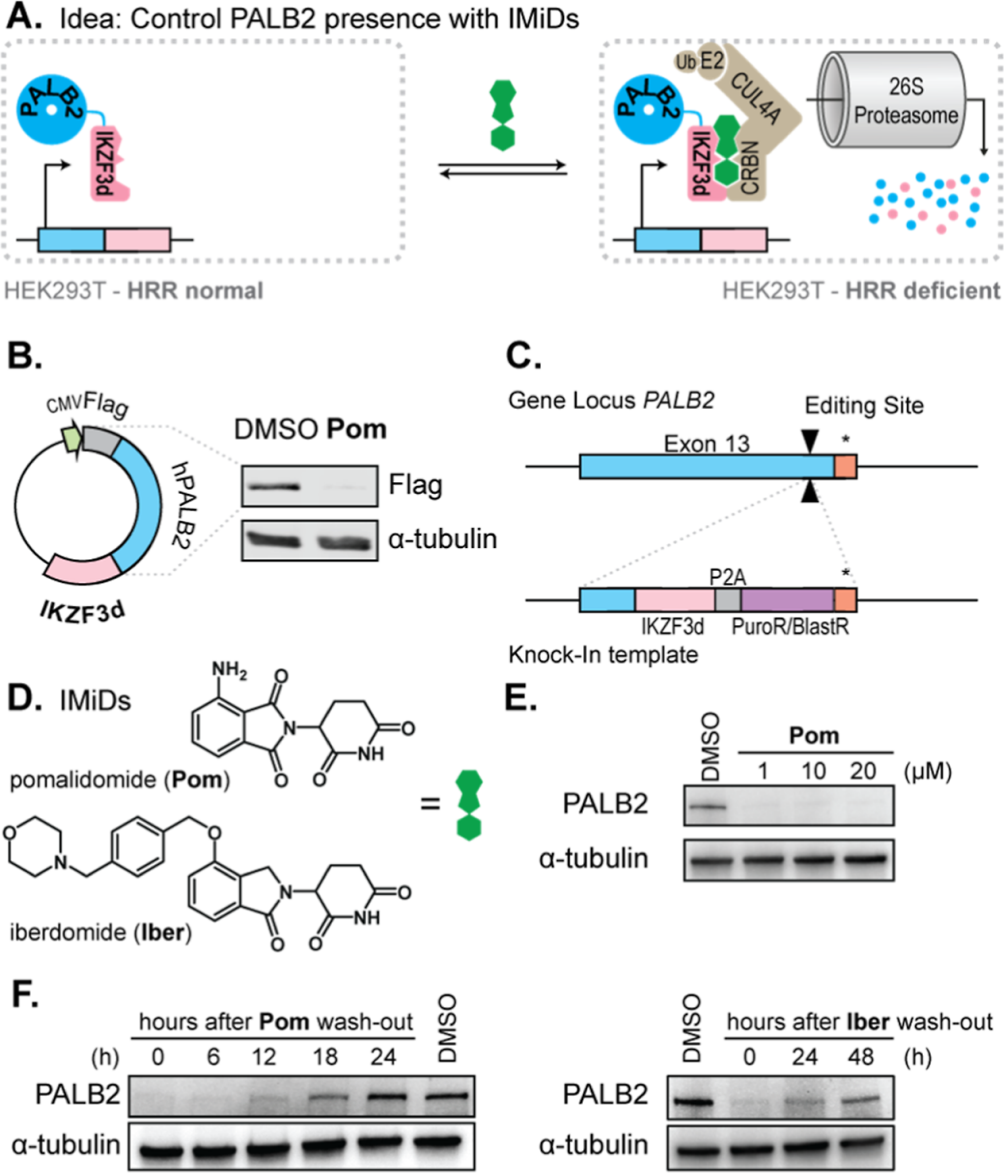
Engineering of PALB2^IKZF 3d/IKZF 3d^. (A) An inducible degron approach to controlling PALB2 levels with immunomodulatory imid drugs (IMiDs). (B) Transient transfection of PALB2-IKZF 3d plasmid in HEK293 cells shows degradation after treatment with 10 μM pomalidomide (**Pom**) for 24 h. (C) Genome editing strategy for targeting the native PALB2 locus. (D) Structure of the IMiD molecules **Pom** and iberdomide (**Iber**) used to induce degradation. (E) **Pom** induces PALB2 degradation in IKZF3d homozygous knock-in HEK293 cells (24 h for each treatment). (F) Wash out experiment of IKZF 3d homozygous knock-in cell line with **Pom** (left) and **Iber** (right). After 24 h treatment of 10 μM **Pom**, medium was removed and cells were washed thrice with PBS. Cells were collected for Western blot after the indicated time points.

To create a cell line in which PALB2 can be inducibly degraded, we aimed to knock in a small-molecule responsive conditional fusion degron at the endogenous PALB2 locus (Figure 3A).^27−31^ Conditional fusion degrons have emerged as powerful tools in target validation because they allow rapid post-translational depletion of a protein, even when a small molecule ligand to the protein is unavailable. In essence, this technology utilizes protein motifs that promote ubiquitination and subsequent proteasomal degradation in the presence of a specific small molecule. We selected the pomalidomide-responsive zinc-finger degron, IKZF3d, due to its reliability and compact size (∼7 kDa).^32^ Given that PALB2 has critical interaction domains at both the N- and C-termini,^33^ we reasoned that the small IKZF3d tag (∼7 kDa),^30^ stood the best chance of retaining PALB2’s function. We first constructed plasmids expressing full-length PALB2 fused N- or C- with the IKZF3d sequence to assess which ones would be functional and degradable. While both N- and C-terminal fusions could be expressed (Figures 3B and S1), degradation upon pomalidomide treatment occurred only with the C-terminal fusion (Figure 3B). Before proceeding with cell line engineering, we next needed to assess whether the critical PALB2/BRCA2 interaction is maintained in the presence of the C-terminal IKZF3d tag. Both coimmunoprecipitation (PALB2-IKZF3d-FLAG overexpression cell lysate, Figure S2A) and a nanobit PPI interaction assay (Figure S2B) confirm the interaction is maintained in cells.^34^

Next, we used CRISPR/Cas9 to knock-in the IKZF 3d degron at exon13 of the PALB2 gene in HEK293 cells (Figure 3C, details in Section S4 of the Supporting Information). Unfortunately, after the first round of editing and selection, we obtained only heterozygous cell lines. Indeed CRISPR genome editing is challenging for proteins involved in HR repair since homology-directed repair is the process by which clean inserts are typically generated.^35,36^ Disrupting HR-related genes often triggers compensatory pathways, such as nonhomologous end joining (NHEJ), making it harder to achieve clean knock-ins. While HR-independent approaches for knock-ins have been developed,^37^ we were fortunate that a second round of editing with a different selection marker delivered several homozygous IKZF 3d knock-in clones (PALB2^IKZF 3d/IKZF 3d^), as confirmed by targeted sequencing (Supporting Information, Section S4). Importantly, both the heterozygous and the homozygous knock-ins showed degradation of PALB2 upon treatment with immunomodulatory imid drugs (IMiDs), and in the case of the homozygous cells PALB2 is barely detectable after degradation. For example, following the addition of **Pom** (Figure 3D) to the culture medium for 24 h, we observed significant degradation of PALB2 (Figure 3E). This degradation could also be induced by other IMiDs, such as iberdomide (**Iber**, Figure 3F, right panel). To confirm that the degradation was driven by the ubiquitination of PALB2-IKZF 3d, we tested whether inhibiting various components of the ubiquitin proteasome pathway (UPP) would rescue PALB2 degradation. Treatment with neddylation inhibitor MLN4924^30^ or 26S proteasome inhibitor MG132^38^ prevented the pomalidomide-induced degradation of PALB2-IKZF 3d (Figure S7B), confirming that drug-induced PALB2 loss relies on the UPP. An advantage of inducible degradation systems in comparison with genetic knockouts is their reversibility. As shown in Figure 3F once **Pom** or **Iber** is removed from the medium the PALB2 levels recover after 24 h. Importantly, incubation for 4 days with **Pom** at high concentrations did not show strong differences between the viability of both WT and KI cell line (Figure S8). With a cell line model for inducible and reversible control of PALB2 levels in hand, we next proceeded to study the impact on HR.

### Pomalidomide Downregulates HRR Function in PALB2^IKZF 3d/IKZF 3d^ Knock-In Cells

**Figure 4:**
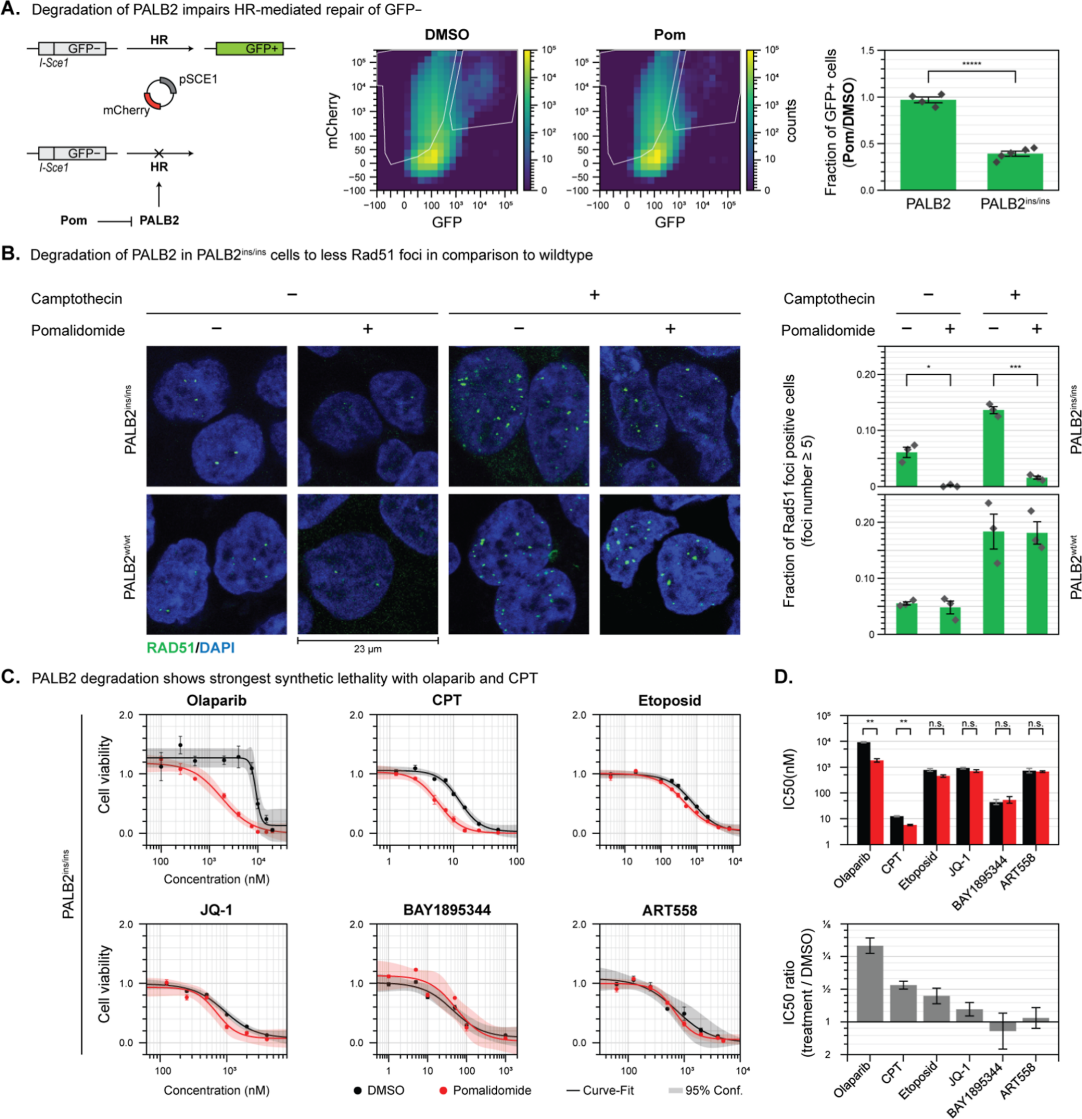
(A) GFP reporter of HR activity to measure effect of PALB2 loss in PALB2^IKZF 3d/IKZF 3d^ cells if treated with **Pom**. (B) RAD51 foci formation to measure cellular response to DNA damage after PALB2 depletion. (C) Testing drug synergies with PALB2 depletion: Cells were pretreated with 10 μM Pomalidomide before DNA damage drugs were also added. Cell viability was measured after 5 days (WST1). (D) Comparison of IC50 with our without pomalidomide. Error bars indicate SEM. Significance tested with a two-sample *t*-test with Welch’s correction. *N* = 3, unless indicated otherwise. * *p* < 0.05, ** *p* < 0.01, *** *p* < 0.001, ***** < 0.000 01.

PALB2 plays a crucial role as a scaffolding protein in HR. Missense variants at different positions in PALB2 can affect its interaction with other key HR proteins, such as BRCA1, BRCA2, and RAD51, leading to impaired HR function.^12,39,40^ To test whether PALB2 degradation would recapitulate the effect of missense mutations in PALB2, we constructed a direct repeat green fluorescent protein (DR-GFP) reporter system in PALB2^IKZF 3d/IKZF 3d^ cells (Figure 4A).^12,41^ This system assesses HRR efficiency by creating a cut in an interrupted GFP open reading frame with an endonuclease, which HRR repairs to give functional GFP, but nontemplated repair processes cannot. Upon treatment with **Pom**, we observed that the number of GFP-positive cells in HEK293 wild-type cells remained largely unchanged compared to the DMSO control group. However, in the PALB2^IKZF 3d/IKZF 3d^ cell line, there was a notable decrease in GFP-positive cells (Figure 4A) upon depletion of PALB2, consistent with compromised HR. These results align with prior work using PALB2 deficient patient cell lines,^39,40,42^ but with a cell-line and treatment protocol that is easy to implement.

We further studied HR impact in a Rad51 foci assay. Rad51 foci form around sites of DNA damage and are a marker of active HR. When we deplete PALB2 using **Pom**, we see that the number of Rad51 foci formed in response to double-strand breaks (DSBs) by camptothecin (**CPT**)^43^ (Figure 4B) significantly decrease. In contrast, compromised Rad51 foci formation is not observed in PALB2 wild-type cells upon **Pom** treatment (Figure 4B). These findings demonstrate that **Pom** treatment in PALB2^IKZF 3d/IKZF 3d^ cells leads to a reduction in HR efficiency, which is independent of any other **Pom** pharmacology.

### Cell Viability Assay with Different DNA Damaging Drugs

We next tested the knock-in (KI) cell line’s viability with different DNA-damaging drugs with and without **Pom** to probe for potential synthetic lethal interactions. In particular, the homozygous KI cell line was depleted of PALB2 by treatment with **Pom** (10 μM, 12 h), followed by addition of different DNA damaging agents. Control treatments were done in wild-type cells, as well as edited cells that were not treated with **Pom**. After 5 days of cotreatment, the survival rate of cells at different concentrations was determined by an end point viability assay (WST1, Figure 4C,D). We tested a total of six drugs, which were carefully selected because of expected synthetic lethal combinations with PALB2.^42,44^ Surprisingly, only the PARP inhibitor olaparib and the DNA topoisomerase inhibitor **CPT** showed noticeable synergistic toxicity. Most previous work on identifying synthetic lethal pairs were performed in cancer cells which might have overlapping types of genome instability, potentially delivering a different sensitivity profile. Hence we plan that future studies with the knock-in cell line will include a comprehensive synthetic lethal pair screen (RNAi or CRISPR), which we can then compare to results from sensitivity screens in cancer cells.

## Discussion

The tools developed here provide a resource for studying PALB2 and HR, while also enabling drug discovery efforts. Whereas traditional assays rely on patient cells with PALB2 loss or siRNA-mediated knockdown of wild-type (WT) PALB2 followed by transient expression of mutated constructs or treatments with small molecules,^42^ our model enables rapid and reversible PALB2 depletion with only a small molecule. For testing PALB2 variants^22,39,40,45^ or drug combinations, the inducible cell line requires only a single transfection step for any variant to be tested or direct treatment with a second small molecule if it is available for the target of interest. By eliminating the labor-intensive nature of conventional knockdown methods and boosting sensitivity, this system significantly reduces the time and effort involved in characterizing emerging variants of uncertain significance or testing synthetic lethal combinations.^12,39,40^

We also present new cellular data indicating that HR loss is surprisingly tolerable in genetically stable HEK cells. This is consistent with CRISPR-based sensitivity profiles across a broad panel of cancer cell lines (downloaded from depmap.org), which show that PALB2 deletion imposes fewer deleterious effects than other high-value oncology targets, such as ATR or CHEK1 (see data from depmap.org in the Supporting InformationSection S7). These findings bolster the idea that therapeutically inhibiting PALB2 would be tolerated and could unmask synthetic lethal vulnerabilities in cells where the DDR is already compromised—such as those with ATM, mismatch repair (MMR), or ATR mutations—or where haploinsufficiency in other HR-related genes (including Fanconi anemia proteins) heightens dependence on remaining DNA repair pathways.^1,2,21,46^ Among core HR factors, PALB2 is notable for possessing a WD40 β-propeller domain^16^ that may be druggable.^17^ RAD51 is the only other core HR factor that has been pursued extensively as a drug target,^18,47−49^ but these efforts have yet to yield potent compounds. BRCA1 and BRCA2 are also challenging to target with small molecules due to their large (and as yet unresolved) structures and their scaffolding function, which makes drug development difficult.^50^ Hence, we posit that PALB2 is the ideal HR protein to test the hypothesis that drugging HR would have exploitable synthetic lethal combinations with certain cancer subtypes.

The reversible cellular model system provides a simple method to study HR and its interaction with other factors, but we further sought a method to precisely track how mutations affect the PALB2–BRCA2 interface. To accomplish this, we built a characterization pipeline that involves biochemical followed by cellular testing. In particular, we employed solid-phase peptide synthesis to study and optimize high-affinity peptides derived from the BRCA2 binding region, systematically refining both the core sequence and peptide length. For assessing the PALB2/BRCA2 interaction in cells we developed a NanoLuc split-luciferase assay. Although the NanoLuc approach does not measure HR outcomes, it excels at quickly confirming direct binding defects and can be scaled for rapid testing of multiple variants.

Collectively, our approaches open new avenues for PALB2 drug discovery. The cell-based depletion model is ideal for functional screens—quickly revealing whether a particular perturbation compromises HR—while the high-affinity peptide and NanoLuc assay information on direct PALB2/BRCA2 binding (which could be used to confirm on–target activity of putative binders). These complementary assays enable rational exploration of PALB2 as a potential therapeutic target. For instance, the tight-binding peptide we describe might be adapted into a cell-permeable agent to test the feasibility of direct PALB2 inhibition. Alternatively, it could serve as a competitive probe in high-throughput screens for small molecules that disrupt the PALB2–BRCA2 interface, paving the way toward novel treatments tailored to exploit synthetic lethal vulnerabilities across diverse cancer genotypes.

## Methods

### Peptide Synthesis

Peptides were synthesized by standard SPPS on a CEM Liberty Blue Discover Bio automated peptide synthesizer on a 0.1 mmol scale with AppTec Rink amide beads (0.53 mmol/g, 100–150 mesh). *N*-Fmoc protected amino acids (0.2 mol/L in DMF) with appropriate side chain protecting groups (Boc, *t*Bu, *Ot*Bu or Trt) were coupled with DIC (1 mol/L in DMF) as the activator and Oxyma (1 mol/L) was the activator. Fmoc removal was performed using piperidine (20% (v/v) in DMF). Peptide were cleaved with 3 mL of a mixture of TFA/water/TIPS/phenol (85:5:5:5 v/v/v/v) and precipitated with cold diethyl ether (500%, v/v). The precipitated peptide was collected and purified by preparative HPLC (Gemini NX-C18, 5 μm, 110 Å, 250·21.2 mm) with water (0.1% v/v TFA) and acetonitrile (0.1% v/v TFA).

### Direct Fluorescent Polarization

FLU-GG-KADLGPISLNWFEELSSEA (Peptide Protein Research Ltd., Fareham, UK) was dissolved in DMSO at 1 μM. PALB2C (22 μM, in 20 mM HEPES, 100 mM NaCl, 1 mM DTT, 1 mM EDTA) was diluted to a final concentration of 4.4 μM (same buffer supplemented with 0.01% (v/v) IGEPAL CA-630). The protein was serially diluted and fluorescent peptide was added to a final concentration of 10 nM. After incubation in the dark at RT for 20 min, fluorescence polarization was measured (ex: 485:20, em: 535:25, 25 °C). *K*_D_ was obtained by fitting the signal to a binding model using the python lmfit package with the nonlinear least-squares method.^51^

### Displacement Polarization Assay

To the mixture above, the dark peptide was added at a final concentration of 10 μM (1000 eq. excess). After incubation in the dark at RT for 20 min, fluorescence polarization was measured (ex: 485:20, em: 535:25, 25 °C).*K*_D_ was obtained by fitting the signal to a competitive binding model described previously^52^ by using the python lmfit package with the nonlinear least-squares method.

### NanoBit Assay

The NanoBit CMV MCS BiBiT-Ready Vector (Promega) was modified to express PALB2 with a C-Terminal SmBit and the BRCA2 N-terminal peptide with a N-terminal LgBit. 5000 HEK293 cells were seeded in each well of a 96-well plate with a white bottom and grown for 24 h with cell medium (DMEM supplemented with 10% FCS at 37 °C with 5% CO_2_). The plasmid (100 ng) was transfected with FuGene HD (Promega, E5911) and incubated for 24 h. Then, Nano-Glo Live cell assay substrate (Promega) was added and the luminescence signal was recorded for 1000 ms. Signal was normalized to empty vector.

### DR-GFP Reporter Assay

DR-GFP plasmid^53^ (a gift from Maria Jasin, Addgene plasmid #26475; RRID/Addgene_26475) was transfected into HEK293 cells with TurboFect (Thermo Scientific) at 70% confluency and selected with hygromycin (0.1 g/L, Invivogen) after 48 h. Transfected cells were moved to a 150 mm cell culture dish and cultured in DMEM medium with hygromycin (0.1 g/L). After obtaining cells carrying the DR-GFP reporter, the cells were treated with **Pom** for 24 h. Cells were then transfected with a modified pCBASceI^53^ (a gift from Maria Jasin, Addgene plasmid #26477; RRID/Addgene_26477, I-SceI was expressed with P2A-mCherry to select for positively transfected cells) to introduce DSB at the I-SceI site in inserted DR-GFP plasmid. Four days after transfection, cells were resuspended in PBS and analyzed by flow cytometry.

### Rad51 Foci Assay

50,000 cells (WT HEK293 or HEK293-PALB2^IKZF 3d/IKZF 3d^) were seeded on coverslips and cultured with **Pom** (10 μM). After 24 h, CPT was was added (10 nM). After 12 h, medium was removed and the coverslip was washed with PBS (3 times) and fixed with formaldehyde (4% in PBS) for 15 min, washed with PBS (3 times) and permeabilized for 10 min (0.25% v/v Triton X-100 in PBS), washed with PBS (3 times) and finally blocked with BSA (1% BSA for 1 h). After removing the BSA-containing PBS, coverslips were incubated with primary antibody (Alexa Fluor 488 Anti-Rad51 antibody [EPR4030(3)] (Abcam, ab309674, RRID/AB_3675862), 1:500 in PBS) for 2 h and washed with PBS (3 times). Then, coverslips were mounted with antifade mountant with DAPI (Invitrogen) and fluorescence images were recorded by confocal microscopy (Leica Point Scanning Confocal SP8). Rad51 foci numbers in each cell were counted with Fiji ImageJ.

## Supporting Information Available

The Supporting Information contains additional figures referenced in the main text, all methods used herein, characterization data, and synthesis procedures of compounds S(PDF)
