# Supplementary material for: Exploring the Potential of Homologous Recombination Protein PALB2 in Synthetic Lethal Combinations
Source: ACS Chem Biol. 2025 Apr 29;20(5):1099–106. doi: 10.1021/acschembio.5c00111 (PMC12090178; doi:10.1021/acschembio.5c00111)
Supplement: Supplementary file 1 — cb5c00111_si_001.pdf [file cb5c00111_si_001.pdf]

Exploring the potential of homologous recombination protein PALB2 in synthetic lethal combinations

Xinyan Lu,[a] Basilius Sauter,[a] Aramis Keller,[a] Saule Zhanybekova, and Dennis Gillingham*[a]

[a] Department of Chemistry, University of Basel, 4056 Basel, Switzerland

*correspondence: dennis.gillingham@unibas.ch

Contents

1 General Methods ... 3
1.1 Cell culture ... 3
1.2 Preparation of cell lysates ... 3
1.3 Transient transfection ... 3
1.4 Gel electrophoresis ... 3
1.5 Western blot ... 4
1.5.1 Transfer ... 4
1.5.2 Antibody staining ... 4
1.5.3 Antibodies ... 4

2 N-terminal IKZF3d degradation assay ... 6
2.1 FLAG-IKZF3d-PALB2 ... 6
2.2 Protein sequences ... 6
2.2.1 FLAG-PALB2-IKZF3d ... 6
2.2.2 FLAG-IKZF3d-PALB2 ... 6

3 PALB2-BRCA2 interaction assays ... 7
3.1 Co-IP ... 7
3.2 NanoBit assay ... 7
3.3 Fusion protein sequences ... 8
3.3.1 FLAG-EGFP-IKZF3d ... 8
3.3.2 SmBit-PALB2 ... 8
3.3.3 SmBit-PALB2-IKZF3d ... 9
3.3.4 BRCA2 (1-40) -LgBit ... 9
3.3.5 BRCA2 (1-50) -LgBit ... 9
3.3.6 BRCA2 (21-39) -LgBit ... 9
3.3.7 BRCA2 (21-39, W31R) -LgBit ... 9

4 CRISPR-Cas9 editing ... 10
4.1 MMEJ-assisted gene knock-in ... 10
4.2 HR-mediated gene knock-in ... 11
4.3 Genotype ... 12

5 Cell treatment 14
5.1 Degradation of PALB2 and UPP rescue assays 14
5.2 Cell viability assay 14

6 Homologous recombination assay 16
6.1 DR-GFP reporter 16
6.2 RAD51 foci assay 16

7 PALB2 CRISPR sensitivity comparison to other HR and high-value oncology targets .17
7.1 Depmap data 17

8 Peptide synthesis 18
8.1 Monomers 18
8.2 Reagents 18
8.3 General procedure 18
8.4 Peptides 19

9 Fluorescent polarization assay 21
9.1 PALB2C origin 21
9.1.1 Insect cell expression 21
9.1.2 HEK293T expression 21
9.2 Direct polarization assay 22
9.3 Displacement polarization assay 22
9.4 Results 23
9.4.1 Alanine scan 23
9.4.2 Other peptides 23
9.4.3 Direct measurements 24

10 Bibliography 25

1 General Methods

1.1 Cell culture

HEK293 (ATCC, CRL-1573, RRID:CVCL_0045) and HEK293FLPin (Thermo Fisher Scientific, R75007, RRID:CVCL_U421) cell lines were used for genome engineering or stable cell-line production. Culture medium contained DMEM, and 10 % FCS. The incubator model was HERAcell 150i (Thermo scientific), set at a temperature of 37 °C, with carbon dioxide concentration maintained at 5 %. Generally, subculturing began after 80 % cell confluence was observed in the T75 culture flask. After 10 passage numbers, the batch of cells needed to be frozen and stored at −80 °C. The freezing medium was Bambanker-Cell Freezing Medium (NIPPON Genetics). The digestion solution used for subculturing is trypsin-EDTA (0.05 %), phenol red (Thermo scientific).

1.2 Preparation of cell lysates

Cells were lysed with RIPA (25 mM Tris-HCl, pH 7.6, 150 mM NaCl, 5 mM EDTA, 1 % NP-40, 1 % sodium deoxycholate, 0.1 % SDS) supplemented with fresh 1X protease inhibitor cocktail (PromegaN and 1X PMSF (Thermo Scientific). Cells were collected by spinning at 10 kcfg (4 °C) and removing the supernatant. Generally, 1 million cells were mixed with 50 µL RIPA lysis buffer. After thoroughly mixing with a 100 µl pipette, the mixture was left on ice for 10 min, mixed again and then left to stand on ice for a further 10 min. Centrifuging at 15 kcfg (4 °C) and collecting the supernatant delivers the soluble protein fraction. DC Protein Assay Kit (Bio-Rad) was then used to measure the protein concentration. To prepare the final sample, blue protein loading dye (NEB) was added to the protein solution and boiled for 5 minutes. After cooling, boiled samples were stored at −20 °C.

1.3 Transient transfection

Transient transfection of FLAG-PALB2-IKZF3d, Crispr/Cas9, Donor DNA and Nanobit assay plasmids was performed. Transfection was started at ~70 % confluence. For transfection, either TurboFect (Thermo-Fisher, for FLAG-PALB2-IKZF3d and Crispr/Cas9, Donor DNA plasmids) or FuGENE HD (Promega, for Nanobit assay) were used according to the manufacturer's instructions.

1.4 Gel electrophoresis

Polyacrylamide gels were run with a Mini-PROTEAN system (Bio-Rad, e.g. 1658004) with either pre-casts (Bio-Rad, 4–20% Mini-PROTEAN® TGX Stain-Free™ Protein Gels, 15 well, 15 µl, #4568096) or hand-casts gels (1.5 mm, 10 or 15 wells). Electrophoresis was run with glycine buffer (25 mM Tris, 192 mM glycine, 0.1% SDS, pH 8.3) at a voltage of 80–100 V. For target proteins with a molecular weight > 130 kDa, we run polyacrylamide gels with a concentration lower than 8%. For target proteins with a molecular weight < 40 kDa, we run polyacrylamide

gels with a concentration of 12%. Gels were run until the loading dye reached the bottom of the gel. As a reference, we used AcuteBand prestained protein ladder with a range of 6.5–270 kDa (Lubio Science, LU5001).

Agarose gels were run with a horizontal electrophoresis system (Bio-Rad, Mini-Sub, e.g. 1704401). The agarose was dissolved in 1X TAE buffer (40 mM Tris base, 20 mM acetate and 1 mM EDTA with a pH of 8.6) in a microwave. After initial cooling, Ethidium bromide is added as a fluorescent dye. The gel was typically run at 80–100 V with time depending on fragment size. As a reference, we used either a 1 kb DNA ladder (NEB, N3232) or a 100 bp DNA ladder (Solis Biodyne, 07-11).

Gels were visualized on a Gel Doc XR+ Gel documentation system (Bio-Rad).

1.5 Western blot

1.5.1 Transfer

For both blotting methods, the membrane was made of nitrocellulose was used (0.45 µm, Amersham Protran Premium Western-Blotting-Membrane, GE10600008).

For proteins < 130 kDa, a semi-dry transfer system was used (Trans-Blot Turbo Transfer System, Bio-Rad, 1704150). As a transfer method, the standard SD program was used.

For larger proteins, wet transfer was used instead (Mini Trans-Blot Cell, Bio-Rad, e.g. 1703935). The membrane and gel were sandwiched between eight layers of filter paper before being placed in the transfer chamber. Transfers were performed at 200 V and for 2.5 h with ice added around the tank. The transfer buffer consisted of 25 mM Tris, 192 mM Glycine, and 20 % methanol (v/v).

1.5.2 Antibody staining

After transferring, the membrane was washed with 1X PBS for 5 min and blocked with blocking buffer (Intercept (TBS) Blocking Buffer, Li-COR, e.g. 927-60001) for 1 h and incubated with primary antibodies overnight at 4 °C. After washing the membrane with PBS, the membrane was then incubated for 1 h with the secondary antibody. Them embrane was washed again, and then incubated with ECL Western Blotting Substrate (Promega) according the the manufacturer’s instructions. Gels were visualized on the Gel Doc XR+ Gel Documentation System (Bio-Rad).

1.5.3 Antibodies

- PALB2
  - Polyclonal Rabbit anti-Human PALB2 Antibody (WB) LS-C409909 (LSBio, LS-C409909, RRID:AB_3675860), diluted 1:1000
  - PALB2 Antibody (Novus Biologicals, NB100-60440, RRID:AB_2158773), diluted 1:1000

    - PALB2 (E9R2W, Rabbit) (Cell Signaling Technology, 30253, RRID:AB_2895010), diluted 1:800
- Anti-BRCA2 antibody (Abcam, ab273157, RRID:AB_3675861), diluted 1:1000
- Monoclonal ANTI-FLAG® M2 antibody (Sigma-Aldrich, F1804), diluted 1:1000
- Anti-alpha Tubulin antibody [DM1A] (Abcam, ab7291, RRID:AB_2241126), diluted 1:1000
- beta Tubulin Loading Control Monoclonal Antibody (BT7R) (Thermo Fisher (Antibody), MA5-16308, RRID:AB_2537819), diluted 1:1000

2 N-terminal IKZF3d degradation assay

2.1 FLAG-IKZF3d-PALB2

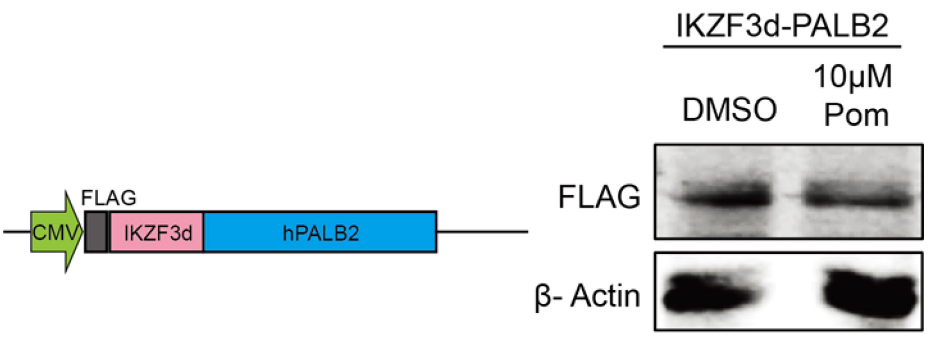

Figure S1. The construct of N-terminal IKZF3d-PALB2 fusion was expressed in HEK293 cells. 24 h treatment of 10 μM Pomalidomide can not induce significant degradation of fusion protein.

2.2 Protein sequences

2.2.1 FLAG-PALB2-IKZF3d

```
MDYKDDDDKVDMDEPPGKPLSCEEKEKLKEKLAFLKREYSKTLARLQRAQRAEKIKHSIKKTVEEQDCLSQQDLSPQLKHSEPKN
KICVYDKLHIKTHLDEETGEKTSITLDVGPESFNPGDGPGGLPIQRTDDTQEHFPHRVSDPSGEQKQKLPSRRKKQQKRTFISQE
RDCVFGTDSLRLSGKRLKEQEEISSKNPARSPVTEIRTHLLSLKSELPDSPEPVTEINEDSVLIPPTAQPEKGVDTFLRRPNFTR
ATTVPLQTLSDSGSSQHLEHIPPKGSSELTTHDLKNIRFTSPVSLEAQGKKMTVSTDNLLVNKAISKSGQLPTSSNLEANISCSL
NELTYNNLPANENQNLKEQNQTEKSLKSPSDTLDGRNENLQESEILSQPKSLSLEATSPLSAEKHSCTVPEGLLFPAEYYVRTTR
SMSNCQRKVAVEAVIQSHLDVKKKGFKNKNKDASKNLNLSNEETDQSEIRMSGTCTGQPSSRTSQKLLSLTKVSSPAGPTEDNDL
SRKAVAQAPGRRYTGKRKSACTPASDHCEPLLPTSSLSIVNRSKEEVTSHKYQHEKLFIQVKGKKSRHQKEDSLSWSNSAYLSLD
DDAFTAPFHRDGMLSLKQLLSFLSITDFQLPDEDFGPLKLEKVKSCSEKPVEPFESKMFGERHLKEGSCIFPEELSPKRMDTEME
DLEEDLIVLPGKSHPKRPNSQSQHTKTGLSSSILLYTPLNTVAPDDNDRPTTDMCSPAFPILGTTPAFGPQGSYEKASTEVAGRT
CCTPQLAHLKDSVCLASDTKQFDSSGSPAKPHTTLQVSGRQGQPTCDCDSVPPGTPPPIESFTFKENQLCRNTCQELHKHSVEQT
ETAELPASDSINPGNLQLVSELKNPSGSCSVDVSAMFWERAGCKEPCIITACEDVVSLWKALDAWQWEKLYTWHFAEVPVLQIVP
VPDVYNLVCVALGNLEIREIRALFCSSDDESEKQVLLKSGNIKAVLGLTKRRLVSSSGTLSDQQVEVMTFAEDGGGKENQFLMPP
EETILTFAEVQGMQEALLGTTIMNNIVIWNLKTGQLLKKMHIDDSYQASVCHKAYSEMGLLFIVLSHPCAKESESLRSPVFQLIV
INPKTTLSVGVMLYCLPPGQAGRFLEGDVKDHCAAAILTSGTIAIWDLLLGQCTALLPPVSDQHWSFVKWSGTDSHLLAGQKDGN
IFVYHYSGTGGSRPFQCNQCGASFTQKGNLLRHIKLH
```

2.2.2 FLAG-IKZF3d-PALB2

```
MDYKDDDDKVDGTGGSRPFQCNQCGASFTQKGNLLRHIKLHGGSMDEPPGKPLSCEEKEKLKEKLAFLKREYSKTLARLQRAQRA
EKIKHSIKKTVEEQDCLSQQDLSPQLKHSEPKNKICVYDKLHIKTHLDEETGEKTSITLDVGPESFNPGDGPGGLPIQRTDDTQE
HFPHRVSDPSGEQKQKLPSRRKKQQKRTFISQERDCVFGTDSLRLSGKRLKEQEEISSKNPARSPVTEIRTHLLSLKSELPDSPE
PVTEINEDSVLIPPTAQPEKGVDTFLRRPNFTRATTVPLQTLSDSGSSQHLEHIPPKGSSELTTHDLKNIRFTSPVSLEAQGKKM
TVSTDNLLVNKAISKSGQLPTSSNLEANISCSLNELTYNNLPANENQNLKEQNQTEKSLKSPSDTLDGRNENLQESEILSQPKSL
SLEATSPLSAEKHSCTVPEGLLFPAEYYVRTTRSMSNCQRKVAVEAVIQSHLDVKKKGFKNKNKDASKNLNLSNEETDQSEIRMS
GTCTGQPSSRTSQKLLSLTKVSSPAGPTEDNDLSRKAVAQAPGRRYTGKRKSACTPASDHCEPLLPTSSLSIVNRSKEEVTSHKY
QHEKLFIQVKGKKSRHQKEDSLSWSNSAYLSLDDDAFTAPFHRDGMLSLKQLLSFLSITDFQLPDEDFGPLKLEKVKSCSEKPVE
PFESKMFGERHLKEGSCIFPEELSPKRMDTEMEDLEEDLIVLPGKSHPKRPNSQSQHTKTGLSSSILLYTPLNTVAPDDNDRPTT
DMCSPAFPILGTTPAFGPQGSYEKASTEVAGRTCCTPQLAHLKDSVCLASDTKQFDSSGSPAKPHTTLQVSGRQGQPTCDCDSVP
PGTPPPIESFTFKENQLCRNTCQELHKHSVEQTETAELPASDSINPGNLQLVSELKNPSGSCSVDVSAMFWERAGCKEPCIITAC
EDVVSLWKALDAWQWEKLYTWHFAEVPVLQIVPVPDVYNLVCVALGNLEIREIRALFCSSDDESEKQVLLKSGNIKAVLGLTKRR
LVSSSGTLSDQQVEVMTFAEDGGGKENQFLMPPEETILTFAEVQGMQEALLGTTIMNNIVIWNLKTGQLLKKMHIDDSYQASVCH
KAYSEMGLLFIVLSHPCAKESESLRSPVFQLIVINPKTTLSVGVMLYCLPPGQAGRFLEGDVKDHCAAAILTSGTIAIWDLLLGQ
CTALLPPVSDQHWSFVKWSGTDSHLLAGQKDGNIFVYHYS
```

3 PALB2-BRCA2 interaction assays

3.1 Co-IP

0.5 million HEK293 cells were seeded per well in a 6-well plate. After 24 h, 2 µg of plasmid pCMV-FLAG-PALB2-IKZF3d or pCMV-FLAG-GFP-IKZF3d (control group) was transiently transfected with TurboFect (Thermo Scientific, e.g. R0531 or R0533) into each well. After 36 h, the medium of at least 3 wells was removed. The cells were washed once with cold PBS on ice. The PBS was drained, and the lysis buffer NETN420 buffer (20 mM Tris–HCl (pH 7.5), 420 mM NaCl, 1 mM EDTA, 0.5 % (v/v) IGEPAL CA-630) was added to each well. A protease inhibitor cocktail (Promega) was added to the lysis buffer before use. After 10 minutes, the liquid from the wells was collected into a centrifuge tube, mixed with a pipette, and let stand on ice for 10 minutes. The suspension was centrifuged at 15 kcfg and the supernatant was collected. Then, anti-FLAG M2-agarose beads (Sigma, M8823) were used to fish for FLAG fusion proteins according to the manufacturer's instructions. The final sample was obtained by boiling the beads together with blue protein sample buffer (NEB, B7703S). The samples were analyzed by Western Blot. The antibodies used were Anti-BRCA2 antibody (Abcam, ab273157, RRID:AB_3675861) and Monoclonal ANTI-FLAG® M2 antibody (Sigma-Aldrich, F1804).

3.2 NanoBit assay

NanoBiT CMV MCS BiBiT-Ready Vector (Promega) was used to construct a plasmid that can express LgBit fusion and SmBit fusion at the same time in cells. On this plasmid, both fusions are under a CMV promoter but with transcription in opposite directions. The two CMV segments share the same enhancer sequence. The BRCA2 N-terminal peptide (B2N) is fused to the N-terminus of LgBit; the PALB2 sequence is fused to the C-terminus of SmBit. Before transfection, HEK293 cells were seeded on a 96-well plate with a white bottom (P8616-50EA, Sigma), with 5000 cells per well. Transfection was initiated 24 hours later with 100 ng plasmid and FuGENE HD (Promega, E5911). 24 hours after transfection, the luminescence signal of each well was obtained by adding Nano-Glo Live Cell Assay System (Promega) and the signal was read on a Tecan Spark 10M (Tecan) for 1000 ms without temperature control.

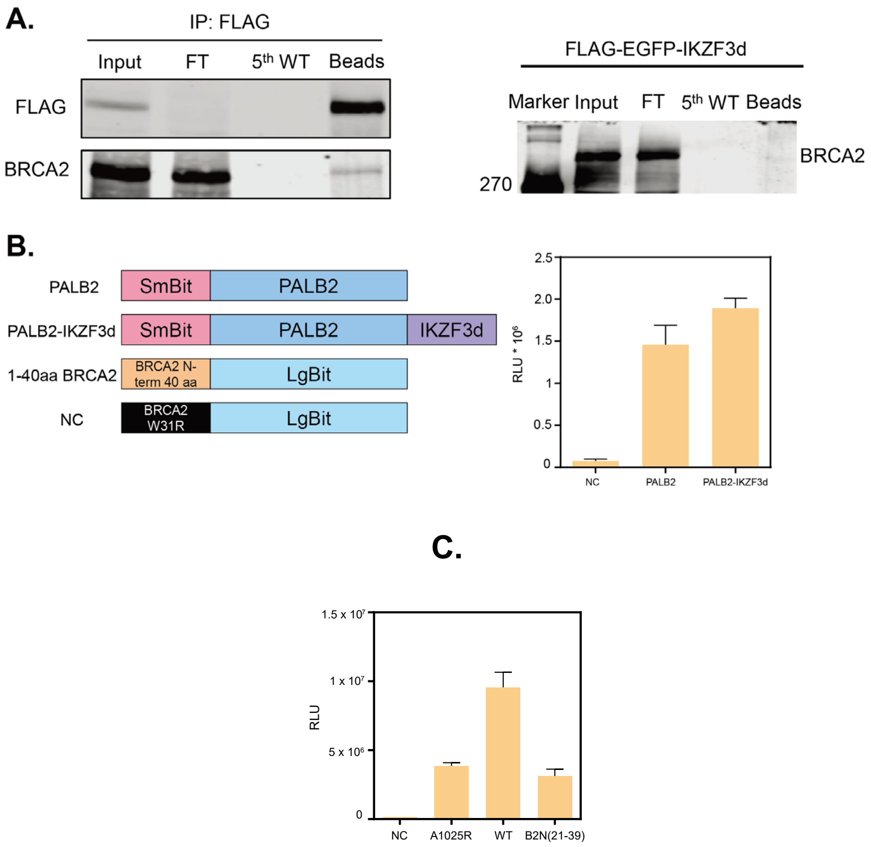

Figure S2. **A)** Co-IP assay of BRCA2 by transient overexpression of FLAG-EGFP-IKZF3d in HEK293 cells. FLAG-magnetic beads were used to pull down fusion protein. Results showed in western blot. FT: flowthrough. 5th WT: washthrough of 5th wash. **B)** Nanobit assay of PALB2-C (835-1186 aa) and PALB2-C-IKZF3d degron fusion. SmBit is fused with PALB2-C or PALB2-C-IKZF3d fusion. LgBit is fused with BRCA2 N-terminal peptide (1-40 aa). Negative control (NC) is produced by fusing a point mutated BRCA2 peptide (21-39 aa of BRCA2, W31R) with LgBit. Result shows that the interaction between PALB2-BRCA2 is not impaired by IKZF3d fusion. **C)** NanoBit interaction results between SmBit-PALB2 key mutant A1025R and BRCA2(1-39)-LgBit. WT represents the wild-type SmBit-PALB2 (835-1186), and the BRCA2 structure that interacts with it is also BRCA2(1-39)-LgBit. B2N (21-39) represents BRCA2 (21-39)-LgBit and is added here as an internal control. The signal ratio is comparable to Figure 2C in the main paper.

3.3 Fusion protein sequences

3.3.1 FLAG-EGFP-IKZF3d

```
MDYKDDDDKVDVSKGEELFTGVVPILVELDGDVNGHKFSVSGEGEGDATYGKLTLKFICTTGKLPVPWPTLVTTLTYGVQCFSRY
PDHMKQHDFFKSAMPEGYVQERTIFFKDDGNYKTRAEVKFEGDTLVNRIELKGIDFKEDGNILGHKLEYNYNSHNVYIMADKQKN
GIKVNFKIRHNIEDGSVQLADHYQQNTPIGDGPVLLPDNHYLSTQSALSKDPNEKRDHMVLLEFVTAAGITLGMDELYKGTGGSR
PFQCNQCGASFTQKGNLLRHIKLH
```

3.3.2 SmBit-PALB2

```
MVTGYRLFEEILGSSGGGGSGGGGSRSVEQTETAELPASDSINPGNLQLVSELKNPSGSCSVDVSAMFWERAGCKEPCIITACED
VVSLWKALDAWQWEKLYTWHFAEVPVLQIVPVPDVYNLVCVALGNLEIREIRALFCSSDDESEKQVLLKSGNIKAVLGLTKRRLV
SSSGTLSDQQVEVMTFAEDGGGKENQFLMPPEETILTFAEVQGMQEALLGTTIMNNIVIWNLKTGQLLKKMHIDDSYQASVCHKA
YSEMGLLFIVLSHPCAKESESLRSPVFQLIVINPKTTLSVGVMLYCLPPGQAGRFLEGDVKDHCAAAILTSGTIAIWDLLLGQCT
ALLPPVSDQHWSFVKWSGTDSHLLAGQKDGNIFVYHYS
```

3.3.3 SmBit-PALB2(A1025R)

MVTGYRLFEEILGSSGGGGSGGGGSRSVEQTETAELPASDSINPGNLQLVSELKNPSGSCSVDVSAMFWERAGCKEPCIITACED
VVSLWKALDAWQWEKLYTWHFAEVPVLQIVPVPDVYNLVCVALGNLEIREIRALFCSSDDESEKQVLLKSGNIKAVLGLTKRRLV
SSSGTLSDQQVEVMTFAEDGGGKENQFLMPPEETILTFAEVQGMQE**R**LLGTTIMNNIVIWNLKTGQLLKKMHIDDSYQASVCHKA

YSEMGLLFIVLSHPCAKESESLRSPVFQLIVINPKTTLSVGVMLYCLPPGQAGRFLEGDVKDHCAAAILTSGTIAIWDLLLGQCT
ALLPPVSDQHWSFVKWSGTDSHLLAGQKDGNIFVYHYS

3.3.4 SmBit-PALB2-IKZF3d

MVTGYRLFEEILGSSGGGGSGGGGSRSVEQTETAELPASDSINPGNLQLVSELKNPSGSCSVDVSAMFWERAGCKEPCIITACED
VVSLWKALDAWQWEKLYTWHFAEVPVLQIVPVPDVYNLVCVALGNLEIREIRALFCSSDDESEKQVLLKSGNIKAVLGLTKRRLV
SSSGTLSDQQVEVMTFAEDGGGKENQFLMPPEETILTFAEVQGMQEALLGTTIMNNIVIWNLKTGQLLKKMHIDDSYQASVCHKA
YSEMGLLFIVLSHPCAKESESLRSPVFQLIVINPKTTLSVGVMLYCLPPGQAGRFLEGDVKDHCAAAILTSGTIAIWDLLLGQCT
ALLPPVSDQHWSFVKWSGTDSHLLAGQKDGNIFVYHYSGTGGSRPFQCNQCGASFTQKGNLLRHIKLH

3.3.5 BRCA2 (1-40) -LgBit

MPIGSKERPTFFEIFKTRCNKADLGPISLNWFEELSSEAPGNSGSSGGGGSGGGGSSGVFTLEDFVGDWEQTAAYNLDQVLEQGG
VSSLLQNLAVSVTPIQRIVRSGENALKIDIHVIIPYEGLSADQMAQIEEVFKVVYPVDDHHFKVILPYGTLVIDGVTPNMLNYFG
RPYEGIAVFDGKKITVTGTLWNGNKIIDERLITPDGSMLFRVTINS

3.3.6 BRCA2 (1-50) -LgBit

MPIGSKERPTFFEIFKTRCNKADLGPISLNWFEELSSEAPPYNSEPAEESGNSGSSGGGGSGGGGSSGVFTLEDFVGDWEQTAAY
NLDQVLEQGGVSSLLQNLAVSVTPIQRIVRSGENALKIDIHVIIPYEGLSADQMAQIEEVFKVVYPVDDHHFKVILPYGTLVIDG
VTPNMLNYFGRPYEGIAVFDGKKITVTGTLWNGNKIIDERLITPDGSMLFRVTINS

3.3.7 BRCA2 (21-39) –LgBit (B2N)

MKADLGPISLNWFEELSSEAGNSGSSGGGGSGGGGSSGVFTLEDFVGDWEQTAAYNLDQVLEQGGVSSLLQNLAVSVTPIQRIVR
SGENALKIDIHVIIPYEGLSADQMAQIEEVFKVVYPVDDHHFKVILPYGTLVIDGVTPNMLNYFGRPYEGIAVFDGKKITVTGTL
WNGNKIIDERLITPDGSMLFRVTINS

3.3.8 BRCA2 (21-39, W31R) -LgBit

MKADLGPISLNRFEELSSEAGNSGSSGGGGSGGGGSSGVFTLEDFVGDWEQTAAYNLDQVLEQGGVSSLLQNLAVSVTPIQRIVR
SGENALKIDIHVIIPYEGLSADQMAQIEEVFKVVYPVDDHHFKVILPYGTLVIDGVTPNMLNYFGRPYEGIAVFDGKKITVTGTL
WNGNKIIDERLITPDGSMLFRVTINS

4 CRISPR-Cas9 editing

4.1 MMEJ-assisted gene knock-in

To create PALB2-IKZF3 degron (IKZF3d) fusion in Flp-In-293 cells (CVCL_U421), the PITCh knock-in system was utilized. In this process, pCRIS-PITChv2-FBL (#63672, Addgene) as a donor DNA carries the puromycin resistance gene for selection. A pair of short homology locating arms and the IKZF3d sequence were integrated into the same donor plasmid. pX330A-FBL/PITCh (#63671, Addgene) expresses the CAS9 nuclease and the sgRNA targeting exon 13 of PALB2. This plasmid also expresses the sgRNA targeting the PITCH sequence of pCRIS-PITChv2-FBL for the formation of donor DNA fragments.

When the confluency of HEK293FLPin cells in a 6-well plate reached ~70 %, 2 µg of the plasmids described above were transiently co-transfected (1:1) into the cells with TurboFect (Thermo Scientific, e.g. R0531 or R0533). After 48 h, the puromycin selection was started. Transfected cells from one well were moved to a 150 mm cell culture dish and cultured in DMEM medium with 0.1 µg/ml puromycin (InvivoGen). Medium was refreshed every three days for two weeks. In the end, 30 colonies were picked for genotyping and sequencing. As a result, only one heterozygous clone (Colony1) was found with the correct IKZF3d knock-in at the end of the PALB2.

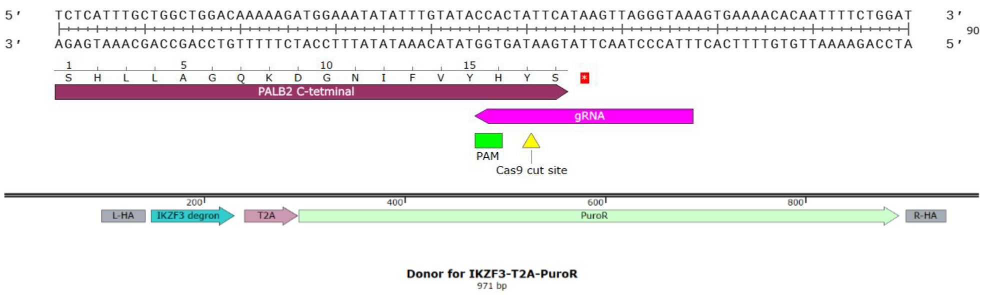

Figure S3. MMEJ knock-in. Sequence of gRNA and donor DNA IKZF3-T2A-Puro.

Sequence of donor DNA for IKZF3-T2A-Puro R 5'-3'

Gray: micro homology arms Blue: IKZF3d Purple: T2A Green: Puro R

TTGACTAGTTATTAATAGTAATCAATTACGGGGTCATTAGTTCATAGCCCATATATGGAGTTCCGCGTTACATAGCATCGTACGC
GTACGTGTTTGGGGCTGGACAAAAAGATGGAAATATATTTGTATACCACTATTCAGGTACCGGCGGATCTCGCCCATTCCAGTGT
AATCAGTGTGGGGCATCTTTTACTCAGAAAGGTAACCTCCTCCGCCACATTAAACTGCACGGAAGCGGAGAGGGCAGAGGAAGTC
TGCTAACATGCGGTGACGTCGAGGAGAATCCTGGACCTATGACCGAGTACAAGCCCACGGTGCGCCTCGCCACCCGCGACGACGT
CCCCCGGGCCGTACGCACCCTCGCCGCCGCGTTCGCCGACTACCCCGCCACGCGCCACACCGTCGACCCGGACCGCCACATCGAG
CGGGTCACCGAGCTGCAAGAACTCTTCCTCACGCGCGTCGGGCTCGACATCGGCAAGGTGTGGGTCGCGGACGACGGCGCCGCGG
TGGCGGTCTGGACCACGCCGGAGAGCGTCGAAGCGGGGGCGGTGTTCGCCGAGATCGGCCCGCGCATGGCCGAGTTGAGCGGTTC
CCGGCTGGCCGCGCAGCAACAGATGGAAGGCCTCCTGGCGCCGCACCGGCCCAAGGAGCCCGCGTGGTTCCTGGCCACCGTCGGC
GTCTCGCCCGACCACCAGGGCAAGGGTCTGGGCAGCGCCGTCGTGCTCCCCGGAGTGGAGGCGGCCGAGCGCGCCGGGGTGCCCG
CCTTCCTGGAGACCTCCGCGCCCCGCAACCTCCCCTTCTACGAGCGGCTCGGCTTCACCGTCACCGCCGACGTCGAGGTGCCCGA
AGGACCGCGCACCTGGTGCATGACCCGCAAGCCCGGTGCCTGATTCGAATAAGTTAGGGTAAAGTGAAAACACAATTTTCTGGAT
ATATCCAAACACGTACGCGTACGATGCTCTAGAATG

4.2 HR-mediated gene knock-in

The Colony1 from PITCh gene editing was heterozygous. To obtain a homozygous IKZF3 degron knock-in cell line, we chose the homologous recombination (HR)-mediated knock-in. Two plasmids were included in the process: a donor DNA plasmid containing a pair of 750 bp homology arms, IKZF3 sequences, and blasticidin resistance gene; a plasmid expressing sgRNA targeting EXON13 of PALB2 and Cas9 nuclease.

The protocol of cell culture, plasmid co-transfection, and antibiotic screening of cell clones are consistent with the PITCh process described above. The concentration of blasticidin for screening was 5 µg/ml. Ultimately, six clones were obtained from the 150 mm culture dish. During the expansion of cell clones, the medium was always maintained with 5 µg/ml blasticidin and 0.1 µg/ml puromycin. After genotyping and sequencing, only one clone was determined were the IKZF3d knock-in was homozygous and the PALB2 sequence remained intact. This homozygous cell line is referenced herein as PALB2$^{IKZF3d/IKZF3d}$.

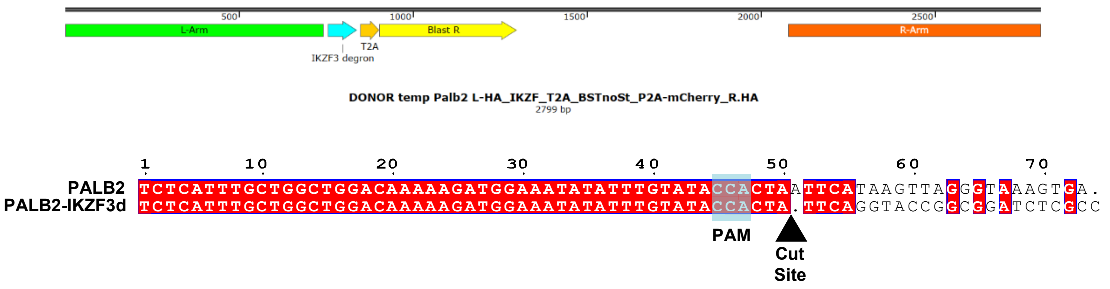

Figure S4. HR knock-in.

Sequence of donor DNA for IKZF3-T2A-Blast R 5'-3'

Gray: homology arms Blue: IKZF3d Purple: T2A Green: Blast R

TCCCACTGAATATGGAAACAGGGTAATCTCAAATAGTGAAAAGTGCAATGAAGATAAAAACATATATGTCATAAAGATGGAACAG
ATCCCGTATGTGTGTGCACACTGCCTGAGATAGAGGATTAGGGGTGACCTTTCTATGGACATAACAGGCCTGAATGATGACAAGA
ACTCAGCCATGCTAAGAGCTGGGGTGAGAGCCTGTCAGGGAGAAAGAATGGCAAGAGCAGTGACACTGAGTTGGGACTGAGTTTG
GCCAGAGTGATGGAGAGTGGAAGGAAGGCCACTGTGCGCCAGCCTTCAATTGCTACAAGATGCATCAGAGAGATTGGCAGGAACT
GGACATGTGATTCTGTCCAAAACTGCAACACAATAGCCAACAGACCTCTAAGGCTACAAATGTAGGCATTCATAGTTACAGGGAT
TTTTGTTCCTGTTGCTGGTTTTGGGAACATGGTTTTGACCTTTTTTTTTTTTTTTAATTGTTTTTTGGATATGTAATCTGAATTAT
ATCTTCTTTGTATGCTATCAGGTTCCTGGAAGGTGACGTGAAAGATCACTGTGCAGCAGCAATCTTGACTTCTGGAACAATTGCC
ATTTGGGACTTACTTCTCGGTCAGTGTACTGCCCTCCTCCCACCTGTCTCTGACCAACATTGGTCTTTTGTGAAATGGTCGGGTA
CAGACTCTCATTTGCTGGCTGGACAAAAAGATGGAAATATATTTGTATACCACTATTCAGGTACCGGCGGATCTGGCGGATCTCG
CCCATTCCAGTGTAATCAGTGTGGGGCATCTTTTACTCAGAAAGGTAACCTCCTCCGCCACATTAAACTGCACGGAAGCGGAGAG
GGCAGAGGAAGTCTGCTAACATGCGGTGACGTCGAGGAGAATCCTGGACCTATGGCCAAGCCTTTGTCTCAAGAAGAATCCACCC
TCATTGAAAGAGCAACGGCTACAATCAACAGCATCCCCATCTCTGAAGACTACAGCGTCGCCAGCGCAGCTCTCTCTAGCGACGG
CCGCATCTTCACTGGTGTCAATGTATATCATTTTACTGGGGGACCTTGTGCAGAACTCGTGGTGCTGGGCACTGCTGCTGCTGCG
GCAGCTGGCAACCTGACTTGTATCGTCGCGATCGGAAATGAGAACAGGGGCATCTTGAGCCCCTGCGGACGGTGCCGACAGGTGC
TTCTCGATCTGCATCCTGGGATCAAAGCCATAGTGAAGGACAGTGATGGACAGCCGACGGCAGTTGGGATTCGTGAATTGCTGCC
CTCTGGTTATGTGTGGGAGGGCTTCGAAGCTACTAACTTCAGCCTGCTGAAGCAGGCTGGAGACGTGGAGGAGAACCCTGGACCT
ATGGTGAGCAAGGGCGAGGAGGATAACATGGCCATCATCAAGGAGTTCATGCGCTTCAAGGTGCACATGGAGGGCTCCGTGAACG
GCCACGAGTTCGAGATCGAGGGCGAGGGCGAGGGCCGCCCCTACGAGGGCACCCAGACCGCCAAGCTGAAGGTGACCAAGGGTGG
CCCCCTGCCCTTCGCCTGGGACATCCTGTCCCCTCAGTTCATGTACGGCTCCAAGGCCTACGTGAAGCACCCCGCCGACATCCCC
GACTACTTGAAGCTGTCCTTCCCCGAGGGCTTCAAGTGGGAGCGCGTGATGAACTTCGAGGACGGCGGCGTGGTGACCGTGACCC

```
AGGACTCCTCCCTGCAGGACGGCGAGTTCATCTACAAGGTGAAGCTGCGCGGCACCAACTTCCCCTCCGACGGCCCCGTAATGCA
GAAGAAGACCATGGGCTGGGAGGCCTCCTCCGAGCGGATGTACCCCGAGGACGGCGCCCTGAAGGGCGAGATCAAGCAGAGGCTG
AAGCTGAAGGACGGCGGCCACTACGACGCTGAGGTCAAGACCACCTACAAGGCCAAGAAGCCCGTGCAGCTGCCCGGCGCCTACA
ACGTCAACATCAAGTTGGACATCACCTCCCACAACGAGGACTACACCATCGTGGAACAGTACGAACGCGCCGAGGGCCGCCACTC
CACCGGCGGCATGGACGAGCTGTACAAGTAATCTAGATAAGTTAGGGTAAAGTGAAAACACAATTTTCTGGATATATTGGGCCTC
TTAGTATTTTTTGGAGTTTTAAATATAAAGGAGAATATCTGAATGACACTTAAAATGATTGCTTGTTTATGTCCAGACAGACTTA
TTTTTTATTCTAATGATGGTAGCACCACTGATCTTGGATGTACATTTATGTATACTTTGAGAAAAAGGGTTTTAGGTTGATTTTT
GTAATTTCCCACATTTGTACATGTGCTTTTAAAGGTGTACATAAAGCTTCAAATGGCAATAAATATTTATTTTTATACATTCTGC
TTGGCATGTTATTGTTTCCCATTCTTTCAAGATCATTTGCAGAAGCAAGAACTAATTATTATACAACCAGGATATTTAATCAATA
GTCTTTGCCTAGTAAGTGTAAATTTCAGTTCAGTTAACTTACTACACTAATATGCAATATACTTTTTGTGCTATTTGTAATACTT
TATTTTTACATACATACTAAAATAAAATGAAACCTACTAAAATATCCTAATTTAGGGATACTCTTACTCTTTTTCATTCTCATGC
ATCTTTCATGCCAGTGCTGCATTATCACTAGAGTCACCTTTTTTTGAGATGGAGCCTCACTGGTCACCCAGGCGGGAGTGCAGTG
GCATGATCTCGGCTCATTGCAACCTCTGCCTCCCAGGTTAAGTGATTCCCCTGCTTCAGCCTCTCAAGTAGCTGGGATT
```

4.3 Genotype

Genotype 1 for WT allele of PALB2 (Below). Primers pair are shown in Table S1. PCR template: WT (genome DNA extracted from WT HEK293 Flpin cells), Colony1 (genome DNA extracted from PITCh product heterozygous colony1), Homozygous KI (genome DNA extracted from second HR knock-in editing cell clone). In Homozygous KI, there is no more band of WT PCR product (~1100bp).

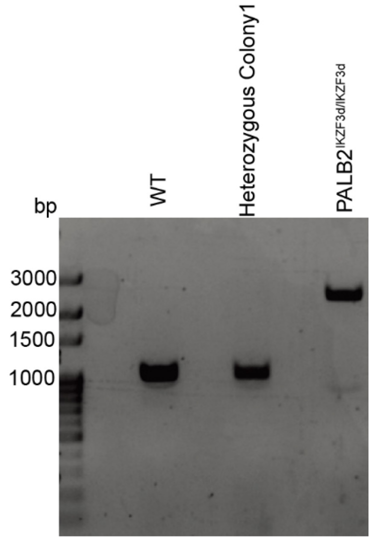

Figure S5. Genotype 1 for WT allele of PALB2. Three PCR reaction used the same primer pair (Table S1).

Table S1. Primers of Genotype1

Description	Sequence 5'→3'	Length
**Genotype1 PCR Forward**	AGGTAGGTTTCTGCTTCATAGATGG	25
**Genotype1 PCR Reversed**	TACACCTTTAAAAGCACATG	20

Genotype 2 for WT and IKZF3d knock-in allele of PALB2 (Below). Primers pair are shown in table S2. PCR template: WT (genome DNA extracted from WT HEK293 Flpin cells), $PALB2^{IKZF3d/IKZF3d}$ (genome DNA extracted from second HR knock-in editing cell clone). Results show that IKZF3d knock-in only exists in $PALB2^{IKZF3d/IKZF3d}$ cell line and $PALB2^{IKZF3d/IKZF3d}$ cell line does not contain WT allele of PALB2.

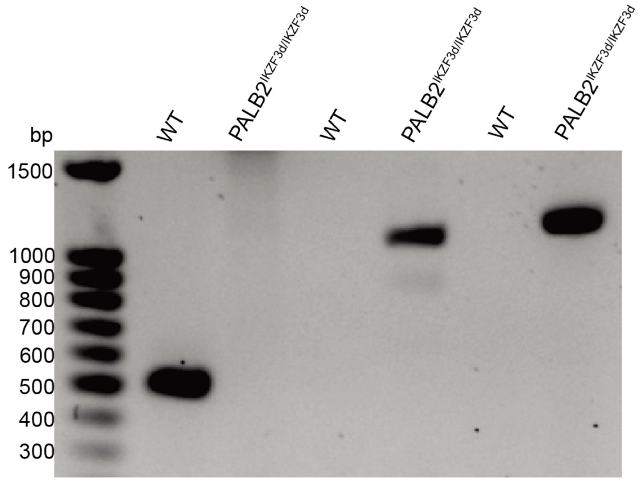

Figure S6. Genotype 2 for WT and IKZF3d knock-in allele of PALB2. From left to right (1-3), three WT / $PALB2^{IKZF3d/IKZF3d}$ parallel PCR shows a comparison between WT genome DNA and homozygous IKZF3 degron knock-in cell line genome DNA: PCR pair 1 shows wt allele without knock-in. PCR pair 2 shows IKZF3-T2A-PuroR knock-in. PCR pair 3 shows IKZF3-T2A-BlastR knock-in.

Table S2. Primers of Genotype 2

Description	Sequence 5'→3'	Length
***Genotype2* PCR1 Forward**	GGATATGTAATCTGAATTAT	20
***Genotype2* PCR1 Reversed**	TACACCTTTAAAAGCACATG	20
***Genotype2* PCR2 Forward**	AGGTAGGTTTCTGCTTCATAGATGG	25
***Genotype2* PCR2 Reversed**	GAGTTCTTGCAGCTCGGTG	19
***Genotype2* PCR3 Forward**	AGGTAGGTTTCTGCTTCATAGATGG	25
***Genotype2* PCR3 Reversed**	ATCGCGACGATACAAGTCA	19

5 Cell treatment

5.1 Degradation of PALB2 and UPP rescue assays

To show PALB2 deegradation, cells were treated with pomalidomide at the indicated concentrations for 24 h. The cells were subsequently lysed and prepared for gel electrophoresis and western blotting.

To show UPP rescue, MG132 and MLN4924 were used to inhibit the ubiquitination-proteasome degradation pathway. The cells were first treated with 1 µM MG132 or 1 µM MLN4924. After 6 h, pomalidomide was added to a final concentration of 10 µM. After 18 h, the cells were collected and lysed.

To show PALB2 recovery, the cells were first treated with 10 µM pomalidomide or 10 µM iberdomide for 24 h. After that, the culture medium was removed, the cells were washed three times with fresh culture medium and then cultured with culture medium without IMiDs. The cells were then collected and lysed.

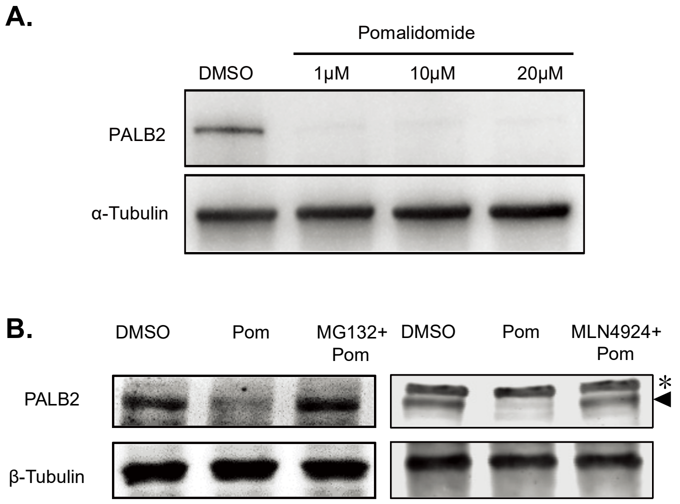

Figure S7. A). Degradation of endogenous PALB2. 10 µM pomalidomide was able to degrade most of the endogenous PALB2 after 24 h. **B).** PALB2 degradation rescue experiment. The cells in the degradation rescue group were first treated with 1 µM MG132 and 1 µM MLN4924 for 6 hours and then treated with 10 µM pomalidomide for 18 hours. Cell samples were collected for WB analysis. The asterisk represents a nonspecific band of PALB2 with this antibody.

5.2 Cell viability assay

In order to search synthetic lethalities of PALB2$^{IKZF3d/IKZF3d}$, cell viability test were run with the Cell Proliferation Reagent WST-1 (Roche).

First, 3000 PALB2$^{IKZF3d/IKZF3d}$ cells were seeded on a 96-well plate. After 16 h, PALB2$^{IKZF3d/IKZF3d}$ cells were treated with 10 µM pomalidomide for 24 hours. After that, the culture medium was

not changed, and DNA damaging reagents of various concentrations were directly added to the culture medium at the indicated concentrations. The control group was set to be pre-treated with DMSO for 24 h, and then DNA damaging reagents of various concentrations were added.

After 5 days, the culture medium of all wells was carefully replaced with fresh medium. Then, 10 µL of WST-1 was added to each well. After incubation in the incubator for 2 hours, the absorbance of each well was measured using a plate reader. Then the decrease in cell viability of each treatment group was calculated based on the absorbance compared with the wells without DNA damaging reagents. As a blank, cell culture medium was used to correct the WST-1 absorbance.

5.3 Cell viability of prolonged degradation of PALB2C

WT and PALB2$^{IKZF3d/IKZF3d}$ cell lines were prepared as described above. The cells were treated with pomalidomide at the indicated concentrations (0, 5, 10 and 40 µM) and cultured for 96 h. The cell viability was measured as described above and visualized in the figure below.

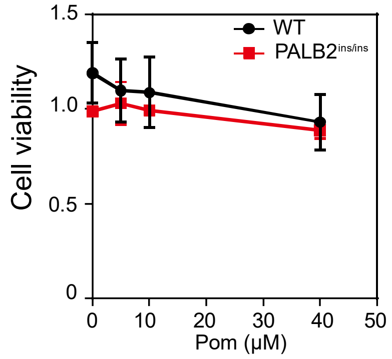

Figure S8. Cell viability of longer pomalidomide treatments. Both cell lines show good tolerance after 96 h even at 40 µM pomalidomide. WT cells are given in black, PALB2$^{IKZF3d/IKZF3d}$ cells in red.

6 Homologous recombination assay

6.1 DR-GFP reporter

DR-GFP[1] substrate (a gift from Maria Jasin, Addgene plasmid #26475; RRID:Addgene_26475) is flanked by human AAVS1 homology arms. Endogenous AAVS1 promoter drives T2A-hygromycin expression after integration. PX458-AAVS1 (#113194, Addgene) encodes sgRNA for human AAVS1 and expresses Cas9 nuclease. The protocol of cell culture, plasmid co-transfection, and antibiotic screening of cell clones was consistent with the PITCh process described before. The concentration of Hygromycin for screening was 0.1 mg/ml (Invivogen). After obtaining cells carrying the DR-GFP reporter, cells were maintained in medium with 10 µM pomalidomide for 24 hours. Cells were then transfected with pCBASceI[1] (a gift from Maria Jasin, Addgene plasmid #26477; RRID:Addgene_26477). This plasmid is an I-SceI endonuclease expression vector with mammalian promoter to introduce a DSB at the introduced I-SceI site. In addition, we also fused I-SceI with P2A-mCherry to indicate the transfection positive level. Four days after transfection, cells were resuspended in PBS and analyzed by flow cytometry using a BD LSR Fortessa.

6.2 RAD51 foci assay

50 000 cells were seeded on coverslips (#354086, Corning) and cultured in medium with 10 µM Pomalidomide. After 24 h, CPT was added in the medium with a final concentration of 10 nM. After 12 h, medium was removed and the cells were gently washed with PBS 3 times. Coverslips were then fixed in 4 % formaldehyde in PBS for 15 min at room temperature. After washing 3 times with PBS, coverslips were permeabilized for 10 min at room temperature in 0.25 % Triton X-100 in PBS. After washing 3 times with PBS, coverslips were blocked with 1 % BSA for 1 h at room temperature. After BSA was removed, coverslips were incubated with primary antibody diluted in 1 % BSA for 2 h at room temperature (Alexa Fluor® 488 Anti-Rad51 antibody [EPR4030(3)] (Abcam, ab309674, RRID:AB_3675862), 1:500) for 2 h at RT. Following primary antibody incubation, coverslips were washed 3 times with PBS. Then, the coverslips were mounted with antifade mountant with DAPI (# P36941, Invitrogen). Fluorescence images were taken by confocal microscopy (LEICA POINT SCANNING CONFOCAL SP8). RAD51 foci numbers in each cell were counted with Fiji ImageJ.

7 PALB2 CRISPR sensitivity comparison to other HR and high-value oncology targets

7.1 Depmap data

CRISPR sensitivity data was visualized as a waterfall plot across all genes and cell models. The highlighted genes are meant to show the general tolerance of cells to loss of HR genes (PALB2, BRCA2, BRCA1, RAD51) and other DDR proteins (PARP1, ATR, RPA1, CHEK1, MDC1). Most cells are not particularly sensitive and PALB2 is less critical than other high-value oncology targets like ATR or CHEK1.

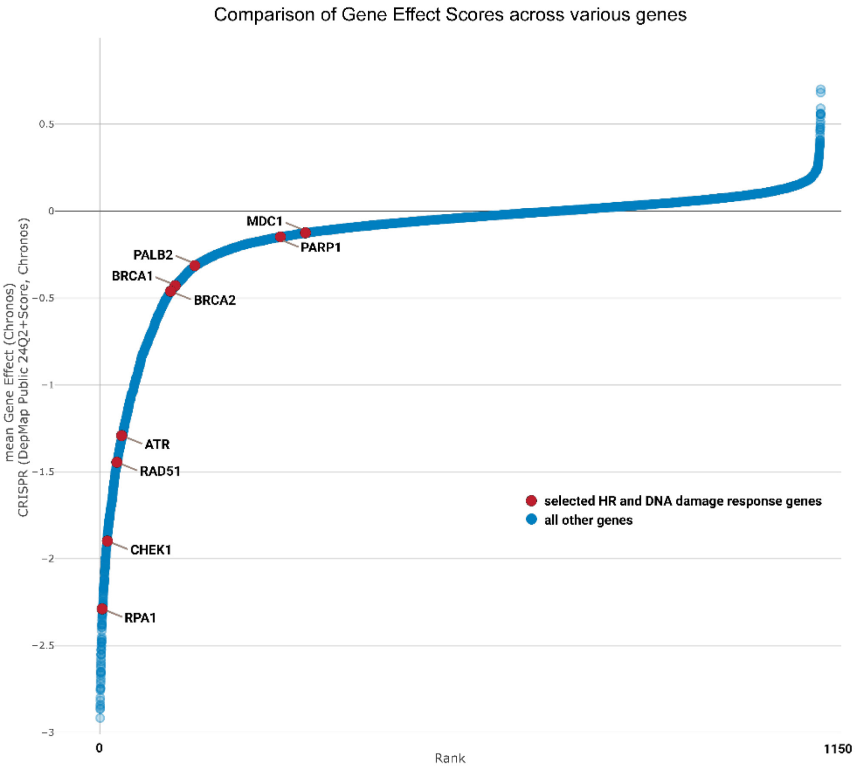

Figure S9. Visualisation of the Demap data. X-Axis is a ranking order, and the y-axis the mean gene effect from a CRISPR screen.

8 Peptide synthesis

8.1 Monomers

All amino acids were employed as their L-enantiomers.

Commercial name	AA-3	AA-1	MW (g mol^{-1})	CAS
**Fmoc-Ala-OH**	Ala	A	311	35661-39-3
**Fmoc-Asn(trt)-OH**	Asn(trt)	N	596	132388-59-1
**Fmoc-Asp(OtBu)-OH**	Asp(OtBu)	D	412	71989-14-5
**Fmoc-Glu(OtBu)-OH**	Glu(OtBu)	E	426	71989-18-9
**Fmoc-Gly-OH**	Gly	G	298	29022-11-5
**Fmoc-Ile-OH**	Ile	I	354	71989-23-6
**Fmoc-Leu-OH**	Leu	L	354	35661-60-0
**Fmoc-Lys(Boc)-OH**	Lys(Boc)	K	468	71989-26-9
**Fmoc-Phe-OH**	Phe	F	388	35661-40-6
**Fmoc-Pro-OH**	Pro	P	338	101555-62-8
**Fmoc-Ser(tBu)-OH**	Ser(tBu)	S	384	71989-33-8
**Fmoc-Trp(Boc)-OH**	Trp(Boc)	W	526	143824-78-6

8.2 Reagents

- AppTec Fmoc-Rink amide AM resin 183599-10-2
- Oxyma 3849-21-6
- N,N''-Bis(isopropyl)carbodiimide (DIC), 99 %, from Apolllo Scientific 693-13-0
- Piperidin from Sigma-Aldrich 110-89-4
- Trifluoroacetic acid (TFA) from ABCR (peptide grade, 99.9%) 76-05-1

8.3 General procedure

Peptides were synthesized using a CEM Liberty Blue Discover Bio automated peptide synthesizer on a 0.1 mmol scale. AppTec Rink amide beads (0.53 mmol/g, 100-150 mesh) served as the solid support. *N*-Fmoc protected amino acids (0.2 M in DMF) with appropriate side chain protecting groups (Boc, tBu, OtBu, Trt) were coupled using DIC (1 M in DMF) as the activator and Oxyma (1 M in DMF) as the activator base. Fmoc deprotection was performed using piperidine (20 % in DMF).
Following synthesis, the resin was transferred to an SPPS syringe equipped with a frit and washed with DCM (3x5 mL). The peptide was cleaved from the resin using a mixture of TFA/H_2O/TIPS/phenol (85:5:5:5 v/v/v/v, 3 mL) for 2 h at room temperature with continuous rotation. The cleaved peptide solution was filtered from the resin and the resin was washed with additional cleavage mixture (2 mL). The combined filtrates were transferred to a 50 mL conical centrifuge tube and precipitated by addition of cold diethyl ether (5 volumes). The suspension was stored overnight at 4 °C.
The precipitated peptide was collected by centrifugation and washed twice with cold diethyl ether. After decanting the supernatant, the pellet was dried at 35 °C for 30 min. The crude

peptide was purified by preparative HPLC (column: Gemini NX-C18, 5 µm, 110 Å, 250 x 21.2 mm; mobile phase: A) water (0.1% (V/V) TFA), B) MeCN (0.1 % (V/V) TFA); gradient: 10 % (5 min) -90 % (40 min) B). Product-containing fractions were identified by HPLC-MS, combined, and lyophilized to afford the peptide as a white solid.

The Cy5-modified peptide was synthesized by modifying the SPPS-synthesised GG-KADLGPISLNWFEELSSEA (P38) still on solid support with 2-azidoacetic acid and HCTU and DIPEA. The Azidopeptide was cleaved from the resin as described above and modified with Cy5-DBCO (Lumiprobe).

8.4 Peptides

Entry	MW [g mol^{-1}]	Peptide Sequence	Pure Sequence
**P1**	1096.16	H-WFEELSSEA-NH2	WFEELSSEA
**P2**	1906.08	H-DLGPISLNWFEELSSEA-NH2	DLGPISLNWFEELSSEA
**P3**	1705.89	H-DLGPISLNWFEELSS-NH2	DLGPISLNWFEELSS
**P4**	721.82	H-WFEEL-NH2	WFEEL
**P5**	949.08	H-LNWFEEL-NH2	LNWFEEL
**P6**	1149.32	H-ISLNWFEEL-NH2	ISLNWFEEL
**P7**	1303.49	H-GPISLNWFEEL-NH2	GPISLNWFEEL
**P8**	1531.74	H-DLGPISLNWFEEL-NH2	DLGPISLNWFEEL
**P9**	1730.99	H-KADLGPISLNWFEEL-NH2	KADLGPISLNWFEEL
**P10**	706.81	H-LNWFE-NH2	LNWFE
**P11**	907.04	H-ISLNWFE-NH2	ISLNWFE
**P12**	1061.21	H-GPISLNWFE-NH2	GPISLNWFE
**P13**	1289.46	H-DLGPISLNWFE-NH2	DLGPISLNWFE
**P14**	1488.72	H-KADLGPISLNWFE-NH2	KADLGPISLNWFE
**P15**	630.75	H-ISLNW-NH2	ISLNW
**P16**	784.92	H-GPISLNW-NH2	GPISLNW
**P17**	1013.17	H-DLGPISLNW-NH2	DLGPISLNW
**P18**	1212.42	H-KADLGPISLNW-NH2	KADLGPISLNW
**P19**	484.6	H-GPISL-NH2	GPISL
**P20**	712.85	H-DLGPISL-NH2	DLGPISL
**P21**	912.11	H-KADLGPISL-NH2	KADLGPISL
**P22**	512.61	H-DLGPI-NH2	DLGPI
**P23**	711.87	H-KADLGPI-NH2	KADLGPI
**P24**	501.59	H-KADLG-NH2	KADLG
**P24**	1673.89	H-AADLGPISLNWFEEL-NH2	AADLGPISLNWFEEL
**P25**	1615.85	H-KADLGPISLNAFEEL-NH2	KADLGPISLNAFEEL
**P26**	1654.89	H-KADLGPISLNWAEEL-NH2	KADLGPISLNWAEEL
**P27**	1672.95	H-KADLGPISLNWFAEL-NH2	KADLGPISLNWFAEL
**P28**	1672.95	H-KADLGPISLNWFEAL-NH2	KADLGPISLNWFEAL

Entry	MW [g mol^{-1}]	Peptide Sequence	Pure Sequence
**P29**	1686.96	H-KAALGPISLNWFEEL-NH2	KAALGPISLNWFEEL
**P30**	1688.88	H-KADAGPISLNWFEEL-NH2	KADAGPISLNWFEEL
**P31**	1744.99	H-KADLAPISLNWFEEL-NH2	KADLAPISLNWFEEL
**P32**	1704.93	H-KADLGAISLNWFEEL-NH2	KADLGAISLNWFEEL
**P33**	1688.88	H-KADLGPASLNWFEEL-NH2	KADLGPASLNWFEEL
**P34**	1714.97	H-KADLGPIALNWFEEL-NH2	KADLGPIALNWFEEL
**P35**	1688.88	H-KADLGPISANWFEEL-NH2	KADLGPISANWFEEL
**P36**	1687.94	H-KADLGPISLAWFEEL-NH2	KADLGPISLAWFEEL
**P37**	1821.11	H-ILKWELDEAFNLFSP-NH2	ILKWELDEAFNLFSP
**P38**	2219.42	H-GG-KADLGPISLNWFEELSSEA-NH2	GGKADLGPISLNWFEELSSEA

9 Fluorescent polarization assay

9.1 PALB2C origin

9.1.1 Insect cell expression

PALB2-C was synthesized by the EPFL protein production facility[1]. In short; the virus was produced and amplified in Sf9 cells. C-terminal His-Tagged 3C-PALB2C was grown in 5 L of Hi5 cells and harvested after 3 days. Pellet was frozen, defrosted and resuspended in 200 mL of wash buffer, supplemented to 5 % glycerol (v/v), 50 µL benzonase, and 2 protease inhibitor tablets. The mixture was clarified with a French press for a total of 4 cycles and centrifuged at 32 kcfg for 1 h. The supernatant was decanted, supplemented with imidazole to a final concentration of 25 mM and added to 5 mL of Ni-NTA beads. The beads were incubated with the supernatant for 1 h.
The beads were washed with 40 column volumes (CV) with wash buffer (500 mM NaCl, 20 mM HEPES, pH 7.5), 10 CV with wash buffer and 50 mM imidazole. The protein was eluted with 6 CV of elution buffer (500 mM NaCl, 20 mM HEPES, 300 mM imidazole, pH 7.5) to a total volume of 38 mL. The elute was concentrated to 1 mL and injected onto a pre-equilibrated Superdex 200 10/300GL column (250 mM NaCl, 10 mM HEPES, 5 mM DTT, 1 mM EDTA, pH 7.5).
A total volume of 950 µL PALBC was received at a concentration of 1 mg/mL (equals 22 µM) in the superdex buffer. This corresponds to a yield of 0.19 mg/L of Hi5 cells.

9.1.2 HEK293T expression

The coding sequence of C terminal part of Palb2 (aa 835-1186) containing the sequence coding HA-tag at 5'end was cloned into pFC14A HaloTag CMV Flexi Vector (Promega). Upon transient expression of resulting construct in HEK293T cells the Palb2-C protein was produced containing HA-tag at N terminus and Halo-tag at C terminus.
HEK293T cells were seeded in eight 10cm Petri dishes (3.5 × 106 cell/dish) in DMEM supplemented with 10% FCS (BioConcept) the day before transfection. Cells were transient transfected (20 ug DNA/dish) using Calcium Phosphate method as described in Addgene protocols.
40 h after transfection the cells were rinsed with Dulbecco's PBS (Sigma), scraped and pelleted by centrifugation at 300 cfg for 4 min. The cell pellet was resuspended in 4 ml of HaloTag purification buffer (PBS, 1 mM DTT and 0.005% IGEPAL-CA630 (Sigma)) supplemented with 1X Protease Inhibitor Cocktail (Promega). The suspension was sonicated on ice using a UP200St (Hielscher) with 30% amplitude, 100% pulse for three rounds of three cycles (10 s on/10 s off, with 1 min rest after each round) on ice.
Cell lysate was harvested by centrifugation at 12 mcfg for 30 min at 4 °C and directly added onto 70 µl (350 µl slurry) pre-equilibrated Magne HaloTag beads (Promega).
The lysate containing HaloTag fusion protein was incubated with the beads overnight at +4°C with constant rotation and washed at room temperature four times with 4 ml purification buffer. Target protein was released from the resin by overnight incubation at +4°C on the thermoshaker (Eppendorf) with 200 µl of proteolytic cleavage buffer containing HaloTEV

[1] https://www.epfl.ch/research/facilities/ptpsp/

(Promega). The resin was washed with another 200 µl of purification buffer at room temperature for 30 min and a second elution was collected. Elution fractions containing the Palb2-C protein were aliquoted and kept at -20°C.
Concentration was measured on a NanoDrop 2000. The concentration was determined to be 8 µM.

MVYPYDVPDYAGSVEQTETAELPASDSINPGNLQLVSELKNPSGSCSVDVSAMFWERAGCKEPCIITACE
DVVSLWKALDAWQWEKLYTWHFAEVPVLQIVPVPDVYNLVCVALGNLEIREIRALFCSSDDESEKQVLLK
SGNIKAVLGLTKRRLVSSSGTLSDQQVEVMTFAEDGGGKENQFLMPPEETILTFAEVQGMQEALLGTTI
MNNIVIWNLKTGQLLKKMHIDDSYQASVCHKAYSEMGLLFIVLSHPCAKESESLRSPVFQLIVINPKTTLSV
GVMLYCLPPGQAGRFLEGDVKDHCAAAILTSGTIAIWDLLLGQCTALLPPVSDQHWSFVKWSGTDSHLL
AGQKDGNIFVYHYSLEPTTEDLYFQ

HA tag
part of the vector with XhoI site
TEV site

9.2 Direct polarization assay

The polarization assay was run according to the method previously described[2]. A fluorescently marked peptide (eg., FLU-GG-KADLGPISLNWFEELSSEA, Peptide Protein Research Ltd., Fareham, UK) was dissolved in DMSO at 1 µM stock solution and diluted with assay buffer to a concentration of 20 nM (20 mM HEPES, 100 mM NaCl, 1 mM DTT, 1 mM EDTA, 0.01 % (v/v) IGEPAL CA-630).
PALB2C was diluted with assay buffer to a final concentration of 4.4 µM in 16 µL. To the other 15 wells, 8 µL of assay buffer was added. PALB2C was then serially diluted. To each of these wells, 8 µL of the fluorescent peptide solution was added.
After incubating for 20 min at room temperature in the dark, fluorescent polarization was measured on a plate reader (excitation at 485:20, emission at 535:25, at 25 °C).
Polarisation curve was fitted to a K_D binding model with a linear transformation using the python lmfit package[3] with the non-linear least squares method.

$$f = \frac{(c_{\mathrm{Prot}} + c_{\mathrm{Probe}} + K_{\mathrm{D}}) - \sqrt{(c_{\mathrm{Prot}} + c_{\mathrm{Probe}} + K_{\mathrm{D}})^2 - 4 \cdot c_{\mathrm{Prot}} \cdot c_{\mathrm{Probe}}}}{2 \cdot c_{\mathrm{Prot}}}$$

$$\mathrm{FP} = a \cdot f + b$$

9.3 Displacement polarization assay

The displacement assay was set up similarly to the direct polarization assay. In addition to the fluorescent prope (always FLU-GG-KADLGPISLNWFEELSSEA), the peptide (1 mM stock in DMSO) was added to a final concentration of 20 µM. This results in final concentrations of 10 µM and 10 nM for the peptide and the probe, respectively.
The resulting binding curve was fitted using the python lmfit package[3] to a linearly transformed competitive K_D binding model described previously[4] using the non-linear least squares method.

$$m = K_{\text{D, Probe}} + K_{\text{D, Pep}} + c_{\text{Probe}} + c_{\text{Pep}} - c_{\text{Prot}}$$

$$n = K_{\text{D, Pep}} \cdot (c_{\text{Probe}} - c_{\text{Prot}}) + K_{\text{D, Probe}} \cdot \left(c_{\text{Pep}} - c_{\text{Prot}}\right) + K_{\text{D, Pep}} \cdot K_{\text{D, Probe}}$$

$$o = -K_{\text{D, Pep}} \cdot K_{\text{D, Probe}} \cdot c_{\text{Prot}}$$

$$\theta = \cos^{-1}\left(\frac{-2m^3 + 9mn - 27o}{2 \cdot \sqrt{(m^2 - 3n)^3}}\right)$$

$$r = 2 \cdot \sqrt{m^2 - 3n} \cdot \cos\frac{\theta}{3} - m$$

$$f = \frac{c_{\text{Probe}} \cdot r}{3 \cdot K_{\text{D, Probe}} + r}$$

$$\text{FP} = a \cdot f + b$$

9.4 Results

9.4.1 Alanine scan

Peptide	N	Concentration (µM)	K_D (µM)	CI (µM)		Start	End	A pos	AA
**P24**	3	10	3.4	2.1	5.9	21	35	21	K
**P29**	3	10	4.6	3.6	5.8			23	D
**P30**	3	10	5.4	4.3	7.1			24	L
**P31**	3	10	5.4	4.1	7.3			25	G
**P32**	3	10	3.6	2.9	4.6			26	P
**P33**	3	10	5.3	4.2	6.6			27	I
**P34**	3	10	4.9	4.2	5.9			28	S
**P35**	3	10	6.7	5.4	8.4			29	L
**P36**	3	10	4.0	3.4	4.8			30	N
**P25**	4	10	5.5	4.8	6.4			31	W
**P26**	1	10	10.1	8.1	13			32	F
**P27**	1	10	5.1	3.8	7.1			33	E
**P28**	1	10	7.0	5.2	9.7			34	E

9.4.2 Other peptides

Peptide	N	Concentration (µM)	K_D (µM)	CI (µM)		Start	End
**P7**	2	10	82.2	36	∞	25	35
**P8**	2	10	7.9	6.1	11	23	35
**P9**	5	10	5.0	4.1	6.3	21	35
**P12**	1	10	17.9	8.3	12	25	33

**P13**	1	10	> 150	120	∞	23	33
**P14**	2	10	25.2	16	54	21	33
**P15**	3	10	> 150	98	∞	27	31
**P16**	3	10	> 150	83	∞	25	31
**P17**	4	10	> 150	140	∞	23.0	31.0
**P22**	1	10	> 150	37	∞	23	27
**P23**	2	10	> 150	89	∞	21	27
**P24**	1	10	> 150	38	∞	21	25

9.4.3 Direct measurements

**Peptide**	**Target Source**	**N**	**Concentration (nM)**	**K_D (µM)**	**CI (µM)**	
**FAM-GG-BRCA(21-39)**	Hi5	20	10	0.63	0.51	0.79
**Cy5-DBCO-GG-BRCA(21-39)**	Hi5	4	10	0.064	0.047	0.086
**FAM-GG-BRCA(10-39)**	Hi5	3	10	0.093	0.081	0.106
**FAM-GG-BRCA(10-39)**	HEK293T	3	10	0.015	0.012	0.018

10 Bibliography

1 Pierce, A. J., Johnson, R. D., Thompson, L. H. & Jasin, M. XRCC3 promotes homology-directed repair of DNA damage in mammalian cells. *Genes & development* **13**, 2633-2638 %@ 0890-9369 (1999).

2 Oliver, A. W., Swift, S., Lord, C. J., Ashworth, A. & Pearl, L. H. Structural basis for recruitment of BRCA2 by PALB2. *EMBO reports* **10**, 990-996-996 (2009). https://doi.org/https://doi.org/10.1038/embor.2009.126

3 lmfit/lmfit-py: 1.2.2 (Zenodo, 2023).

4 Wang, Z.-X. An exact mathematical expression for describing competitive binding of two different ligands to a protein molecule. *FEBS Letters* **360**, 111-114 (1995). https://doi.org/https://doi.org/10.1016/0014-5793(95)00062-E
